# A Systematic Review and Bayesian Network Meta-Analysis Comparing In-Person, Remote, and Blended Interventions in Physical Activity, Diet, Education, and Behavioral Modification on Gestational Weight Gain among Overweight or Obese Pregnant Individuals

**DOI:** 10.1016/j.advnut.2024.100253

**Published:** 2024-06-13

**Authors:** Hongli Yu, Mingmao Li, Guoping Qian, Shuqi Yue, Zbigniew Ossowski, Anna Szumilewicz

**Affiliations:** 1 College of Physical Education, Sichuan University of Science & Engineering, Zigong, Sichuan, China; 2 Department of Fitness, Gdansk University of Physical Education and Sport, Gdansk, Poland

**Keywords:** pregnancy, gestational weight gain, physical activity, diet, lifestyle

## Abstract

**Background:**

Despite the well-documented adverse outcomes associated with obesity during pregnancy, this condition remains a promising modifiable risk factor.

**Objectives:**

The aim of this study was to ascertain the most effective treatment modalities for gestational weight gain (GWG) in pregnant women classified as overweight or obese.

**Methods:**

A systematic search was conducted across 4 electronic databases: Embase, EBSCOhost, PubMed, and Web of Science. To assess the quality of evidence, the Confidence In Network Meta-Analysis (CINeMA) approach, grounded in the Grading of Recommendations Assessment, Development, and Evaluation framework, was employed. A Bayesian network meta-analysis was conducted to synthesize the comparative effectiveness of treatment modalities based on GWG outcomes.

**Results:**

The analysis incorporated 60 randomized controlled trials, encompassing 16,615 participants. Modes of intervention administration were classified as remote (R: eHealth [e] and mHealth [m]), in-person (I), and a combination of both (I+R). The interventions comprised 5 categories: education (E), physical activity (PA), dietary (D), behavior modification (B), and combinations thereof. The quality of the evidence, as evaluated by CINeMA, ranged from very low to high. Compared to the control group, the I-D intervention (mean difference [MD]: −1.27; 95% confidence interval [CI]: −2.23, −0.32), I-PADB (MD: −0.60, 95% CI: −1.19, −0.00), and I-B (MD: −0.34, 95% CI: −0.57, −0.10) interventions showed significant efficacy in reducing GWG.

**Conclusions:**

Preliminary findings suggest that the I-D intervention is the most efficacious in managing GWG among pregnant women who are overweight or obese, followed by I-PADB and I-B+R-B(m) treatments. These conclusions are drawn from evidence of limited quality and directness, including insufficient data on PA components used in the interventions. Owing to the absence of robust, direct evidence delineating significant differences among various GWG management strategies, it is tentatively proposed that the I-D intervention is likely the most effective approach.

This study was registered with PROSPERO as CRD42023473627.

**Statement of Significance**

This study presents new methods using systematic review and Bayesian meta-analysis, combined with direct and indirect evidence, to evaluate effective interventions for managing gestational weight gain in overweight and obese pregnant women. This approach addresses prior gaps by assessing the effectiveness and administration processes of various intervention methods, providing more comprehensive analyses for this specific group of pregnant women.

## Introduction

Obesity has emerged as a significant global public health issue. According to researchers, its occurrence during pregnancy significantly increases the likelihood of adverse health outcomes [1]. Pregnant women with excess weight face a heightened risk of complications, including gestational diabetes, hypertension, preeclampsia, and a greater necessity for cesarean section deliveries [2]. Moreover, their newborns are more likely to have excess birth weight, potentially leading to various health issues in both infancy and later life [3]. A marked increase in excessive gestational weight gain (GWG) among pregnant women has been observed globally in recent years. Notable prevalence rates include 30% in China, 20% in urban India, 50.7% in Australia, and 30% in Brazil [[4], [5], [6]]. Such weight gain can be attributed to factors such as sedentary lifestyles, dietary changes [7], and societal influences, including socioeconomic status, health care accessibility, and cultural norms [8]. Quantitative studies suggest that interventions such as physical activity (PA), behavior modification, and healthy dieting can mitigate the risk of pregnancy-related complications from excessive GWG [[9], [10], [11], [12]]. Consequently, various intervention strategies targeting pregnancies complicated by obesity have emerged, encompassing PA, dietary changes, comprehensive lifestyle counseling, and different administration modes, including face-to-face (FTF), remote (e.g., mHealth and eHealth), and blended approaches [13,14]. However, research on effective interventions and their modes of administration for managing overweight and obese pregnancies remains limited and contentious.

Previous systematic reviews have supported the effectiveness of the FTF mode, combined with various interventions, in improving GWG, metabolic equivalent, and maximal oxygen consumption in overweight and obese pregnant women [[9], [10], [11],^15^]. Nonetheless, FTF modes have limitations, including resource intensity, scheduling challenges, and geographical limitations [16]. Remote administration modes, such as electronic health (eHealth, which refers to the utilization of emerging information and communication technology, particularly the Internet [e.g., websites, short message services, emails, and forums], aimed at improving or facilitating health and health care) [17] and mobile health (mHealth, the provision of health care services through mobile communication devices, such as mobile apps for supervision, monitoring, and management) [18], represent a significant shift in health care delivery, leveraging digital technology to enhance access and efficiency [19]. These modes have shown effectiveness in addressing unhealthy lifestyle behaviors in overweight and obese children and adults [[20], [21], [22]]. The COVID-19 pandemic underscored the utility of telemedicine in providing scalable and flexible health care solutions [19]. However, some research suggests that remote interventions may be less effective than FTF due to challenges such as limited access, digital literacy, and privacy concerns [22,23].

Moreover, systematic reviews have verified the effectiveness of single interventions, including PA, behavior, and dietary approaches, in pregnant women [10,11,24]. Although research on multiple treatments for overweight/obese children and adolescents is extensive, studies focusing specifically on pregnant women are scarce. The evidence for the efficacy of behavior interventions in reducing GWG among overweight or obese pregnant women is mixed. Some meta-analyses have shown significant reductions in GWG based on the Institute of Medicine (IOM) recommendations on GWG, whereas others found no substantial improvements [9,11,25]. These inconsistencies underscore the necessity for a comprehensive and up-to-date meta-analysis to gather reliable evidence. Traditional meta-analyses are limited in their ability to assess the efficacy of single treatments due to data availability constraints. To date, no extensive studies have examined the most effective intervention methods in conjunction with specific administration modes for treating excessive GWG in overweight or obese pregnant women. Furthermore, evidence-based medical studies and national guidelines have yet to conclusively address this complex issue. Therefore, this study aimed to identify the most effective intervention methods for managing GWG in overweight and obese pregnant women by conducting a systematic review and Bayesian meta-analysis using both direct and indirect evidence.

## Methods

### Protocol

Our methodology follows the protocols outlined in the Preferred Reporting Items for Systematic Reviews and Meta-Analyses extension for Network Meta-Analyses (PRISMA-NMA) within the health care sector. Additionally, it aligns with the Cochrane Collaboration guidelines. Other scholars have elucidated these guidelines in their seminal works [26,27]. For enhancing transparency and reproducibility, the PRISMA-NMA checklist has been detailed in the supplementary materials, specifically in Supplemental Table 1. Furthermore, the protocol for this network meta-analysis (NMA) was registered with PROSPERO with the registration number CRD42023473627, thereby adhering to practices for systematic review registration and reporting.

### Search strategy and study selection

A comprehensive search of 4 electronic databases (Web of Science, EBSCOhost, PubMed, and Embase) was conducted by 2 independent researchers from inception until 30 June, 2023. The search strategy was aligned with the Population, Intervention, Comparator, Outcomes, and Study design (Population: pregnant women who are overweight or obese; Intervention: dietary, PA, educational, and lifestyle behavior modification; Comparator: standard care; Outcomes: GWG; and Study design: randomized controlled trials [RCTs]). The search utilized keywords combined with Boolean operators: (pregnancy OR pregnant women OR maternal OR mother) AND (overweight OR obese OR obesity OR excessive weight OR adiposity) AND (RCTs OR random control trials) AND (intervention OR program OR treatment OR management OR education). The detailed search strategy is included in the supplementary material (Supplemental Table 2). A manual search of relevant systematic reviews, major international conferences, meta-analyses, and references to included studies was conducted to ensure comprehensive coverage. The preliminary search results underwent a meticulous screening process, conducted independently by 2 investigators whose identities were mutually concealed to ensure objectivity. This screening was facilitated through Endnote software (version X9, Thompson ISI ResearchSoft). During this phase, any instances of duplicate entries were systematically identified and excluded from further consideration, thereby enhancing the robustness of the dataset under review. Full-text screening for eligibility was subsequently performed by 2 blinded investigators, with conflicts resolved through discussion or consultation with a third expert.

### Inclusion and exclusion criteria

We delineated the eligibility criteria for conducting an NMA by examining various key parameters, including population, interventions, comparator, outcomes, and types of studies. The inclusion criteria were:

1. Obese or overweight pregnant women without additional medical conditions.
2. Interventions, including PA, dietary, educational programs, behavior modification, or combinations thereof, delivered in-person or via eHealth/mHealth.
3. Comparisons between treatments or a control group.
4. Availability of GWG outcome data.
5. RCTs, either parallel or crossover designs.

No restrictions were placed on gestational trimester, geography, ethnicity, language, or publication year. The classification criteria for in-person, eHealth, and mHealth interventions were delineated according to their respective definitions.

Exclusions were: *1*) non-human studies; *2*) interventions outside PA, dietary, behavior modification, or education; *3*) inaccessible data; and *4*) protocols or non-RCTs.

### Data collection and management

Data from the included RCTs were extracted independently by 2 blinded researchers using a standardized form based on the Cochrane Consumers and Communication Review Group guidelines [28]. The extracted data included the primary author's name, age (mean or median), publication year, gestational week, sample size, intervention characteristics, and GWG outcomes at baseline and final observation. Discrepancies in data extraction were resolved through discussion or third-party expert adjudication.

### Quality appraisal

The assessment of methodological quality in the included RCTs was conducted utilizing the Cochrane Collaboration Risk of Bias (ROB) Tool, as delineated by the study [26]. This evaluation process was independently undertaken by 2 researchers for each RCT. The criteria set forth in the Cochrane Manual 5.1.0, which are primarily centered on research bias, encompass 7 distinct aspects: *1*) the generation of a random sequence; *2*) the concealment of allocation; *3*) the blinding of participants and personnel; *4*) the blinding of outcome assessments; *5*) the incompleteness of outcome data; *6*) the selective reporting of results; and *7*) the presence of other potential biases. The literature under analysis was appraised against these criteria and then classified into 1 of 3 categories: “low risk,” “high risk,” or “unclear.” Initially, each literary piece underwent an independent evaluation by 2 researchers, with a third arbitrator intervening to resolve any disagreements pertaining to inclusion or exclusion. The process of evaluating the ROB was conducted using RevMan (version 5.4; Cochrane Collaboration).

Furthermore, the Confidence In Network Meta-Analysis (CINeMA) approach was employed as a means to appraise the quality of the evidence. This approach is grounded in the Grading of Recommendations Assessment, Development, and Evaluation framework, as expounded by other investigators [29,30]. The assessment encompassed 6 domains: within-study bias, reporting bias, imprecision, indirectness, inconsistency, and incoherence. This exhaustive evaluation coalesced the quality of evidence, underpinning the comparative effectiveness of each intervention relative to the control group. This was in alignment with the objectives of the NMA.

Initially, the comparisons were categorized as high-quality evidence. Subsequent re-evaluations considered 5 factors: ROB, imprecision, inconsistency, indirectness, and reporting bias. Each factor was scrutinized for potential concerns or risks that might necessitate downgrading: no concerns or low risk (entailing no downgrade), some concerns (warranting a 1-level downgrade), or major concerns (necessitating a 2-level downgrade). The ultimate grading for each comparison was contingent on the aggregate score across these domains. This categorizes them into 1 of 4 levels of quality: high, moderate, low, and very low.

### Statistical analysis

To ascertain the effect sizes pertaining to successive outcomes, each estimated weighted mean difference (MD) was juxtaposed using the group mean and SD derived from individual studies. The 95% confidence interval (CI) and pooled MD were calculated for the independent assessment of pooled effect sizes. A network map was delineated to visually illustrate both direct and indirect comparisons among various treatments. Regarding the analysis of global inconsistency in each pairwise treatment comparison, a comprehensive global inconsistency model analysis was undertaken. *P* > 0.05 was indicative of nonsignificant global inconsistency. The extent of global inconsistency was gauged using *I*^2^ statistical values. Thresholds were set at 25% for low heterogeneity, 50% for moderate heterogeneity, and 75% for high heterogeneity. This was done following the work of other authors [31]. Local inconsistency was evaluated through the node-splitting method, where *P* < 0.05 denoted local inconsistency.

Network transitivity assessment, a pivotal premise in NMA [32], plays an instrumental role in guiding further analysis in this research. The clinical and methodological characteristics of all incorporated studies, encompassing participant attributes and experimental design, were scrutinized to ascertain comparability across different treatment comparisons. The surface under the cumulative ranking (SUCRA) curve yields a succinct numerical statistic, the cumulative ranking probability plot, summarizing the efficacy of individual treatments. Elevated SUCRA values imply a greater likelihood of treatment efficacy, whereas lower values suggest diminished effectiveness [33]. To detect potential publication bias, an asymmetric funnel plot was constructed. Additionally, loop inconsistencies were scrutinized to affirm the robustness and reliability of the synthesized evidence. The inconsistency factor and the 95% CI were employed to detect loop inconsistency; the inclusion of the value 0 within the 95% CI signifies nonsignificant loop inconsistency. An inconsistency factor proximal to 0 indicates a reduced probability of loop inconsistency [34]. The statistical analyses for the NMA were executed using Stata/SE 17.0 (Stata Corp). All indicators were analyzed utilizing random-effects models, which necessitated the application of the “MVMETA” and “Network” packages.

## Results

### Search results and study characteristics

**FIGURE 1:**
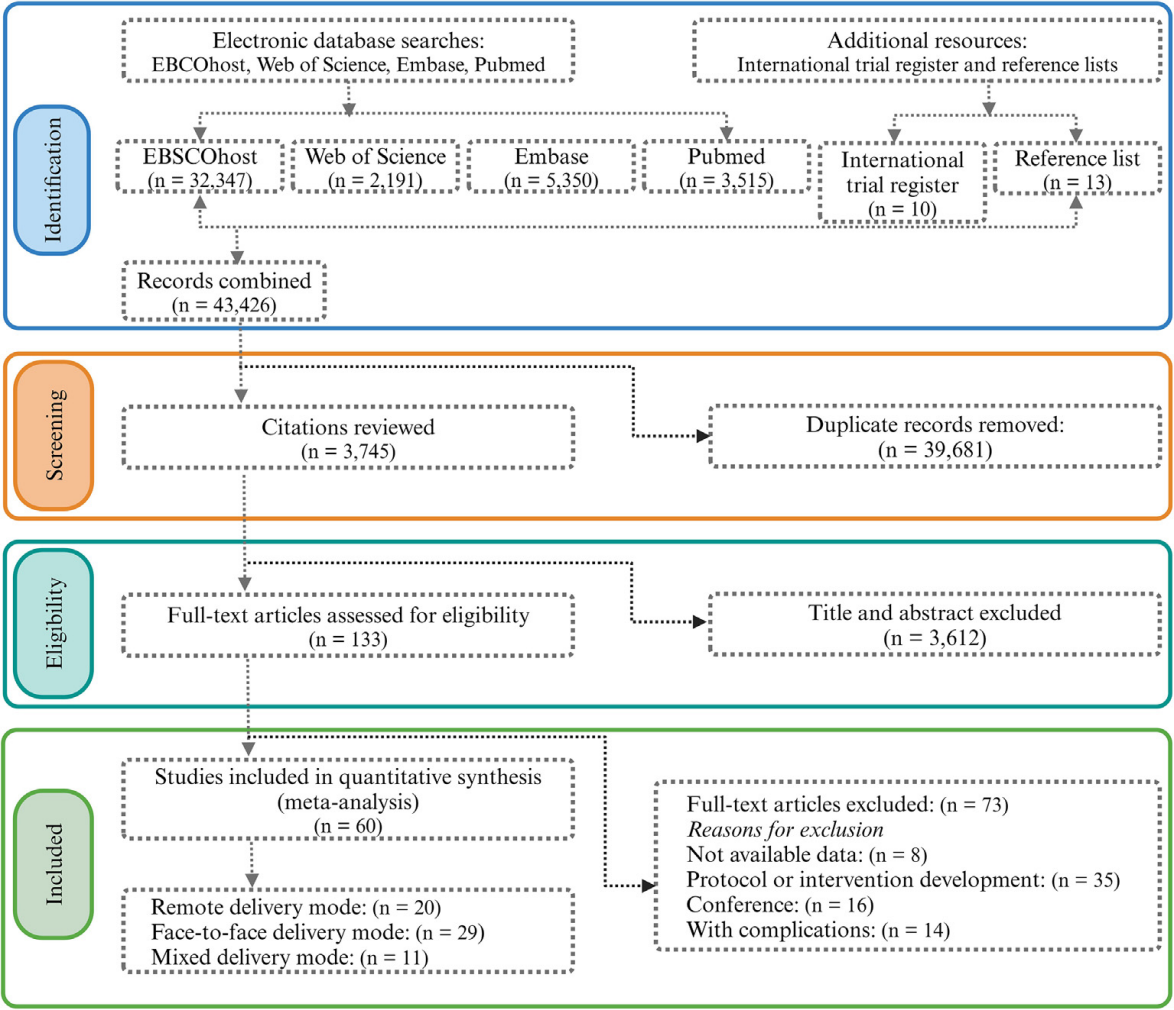
The PRISMA flow diagram delineates the meticulous procedure employed for the selection of articles included in this review.

The PRISMA-NMA flowchart for literature selection is depicted in Figure 1. Initially, 43,426 publications were identified, supplemented by an additional 23 from reference lists and international trial registers. Subsequent to the removal of 39,681 duplicates, 3612 articles were excluded after title and abstract evaluation. A comprehensive full-text review of 133 articles potentially meeting the eligibility criteria was conducted. Of these, 73 were excluded for various reasons: 8 due to lack of data, 35 related to protocol or intervention development, 6 were conference papers, and 14 involved participants with additional diagnosed diseases or disorders. Sixty studies fulfilled the inclusion criteria (see **Supplementary list of included studies**), encompassing a total of 16,615 pregnant women, divided into 8756 in the experimental group and 7859 in the control group, with ages ranging from 20 to 35 y. These included RCTs, published in English between 2002 and 2023, that addressed interventions from early pregnancy to birth and employed remote, in-person, and blended administration methods. Table 1 summarizes the primary data from all 60 studies, with Supplemental Table 3 providing detailed participant and intervention characteristics (refer to Supplementary list of included studies for references).

**TABLE 1:** The demographic characteristics of the study populations and participants deemed eligible for inclusion in this analysis

Characteristics of the 60 included studies	No. (%) of studies
Publication years	
2000–2005	2 (3.3%)
2006–2010	8 (13.3%)
2011–2015	22 (36.7%)
2016–2020	17 (28.4%)
2021–2023	11 (18.3%)
Baseline age (y)	
20–25	2 (3.3%)
26–30	28 (46.7%)
31–35	30 (50%)
Baseline gestation trimester	
Trimester 1	11 (18.3%)
Trimester 2	40 (66.7%)
Trimester 3	8 (13.3%)
Not reported	1 (1.7%)
Total sample size (*n*)	
1–100	31 (51.7%)
101–500	21 (35%)
501–1000	2 (3.3%)
≥1001	6 (10%)
Participant types	
Overweight	5 (8.3%)
Obesity	16 (26.7%)
Overweight and obesity	39 (65%)
Delivering setting	
Face-to-face model	33 (55%)
Remote model	16 (26.7%)
Mixed model	11 (18.3%)
Behavior targets	
Education	2 (3.3%)
Physical activity	9 (15%)
Dietary	1 (1.7%)
Behavior	20 (33.3%)
Multiple	28 (46.7%)
Intervention length (wk)	
≤ 10	2 (3.3%)
11–20	23 (38.3%)
21–30	31 (51.7%)
31–40	4 (6.7%)

### Quality evaluation

The analysis incorporated only RCTs. Regarding random sequence generation, 78.3% (*n* = 47) exhibited a low ROB. Allocation concealment bias was of some concern in 35% of the studies (*n* = 15). A high risk of performance bias, particularly in blinding participants and personnel, was observed in 20% of studies (*n* = 12). Attrition bias was notably high in 10% of the studies (*n* = 6), and 3.3% (*n* = 2) demonstrated a high risk of reporting bias. Other biases, primarily related to small sample sizes, were of some concern in 21.7% (*n* = 13). The ROB assessments for both individual and overall studies are detailed in Supplemental Figures 1 and 2. The evidence quality, evaluated using the CINeMA approach, varied from very poor to high (Supplemental Table 4).

### Network plot

**FIGURE 2:**
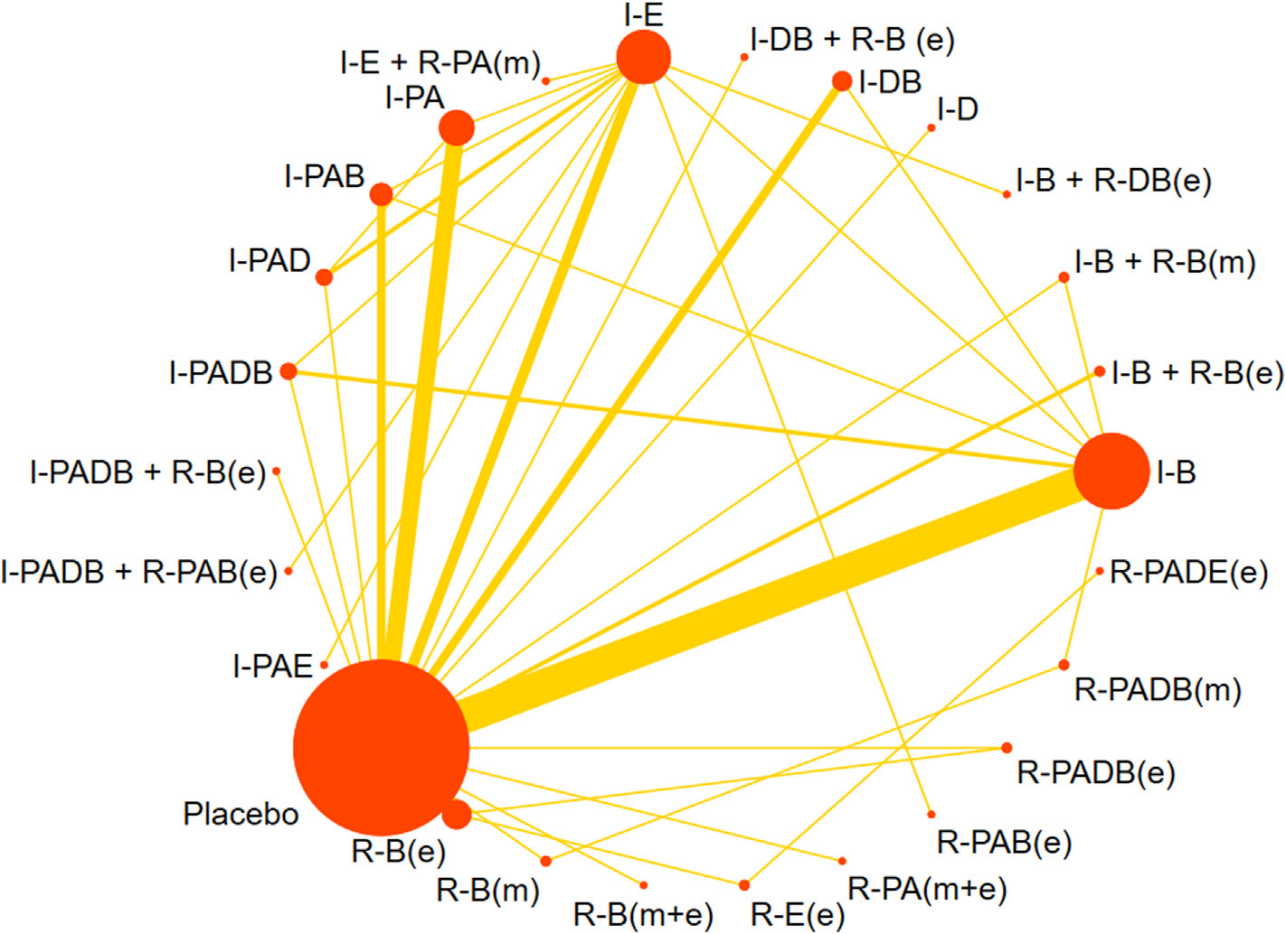
A network diagram, elucidating the direct evidence derived from all intervention arms, focuses on gestational weight gain. In this diagram, the magnitude of each node correlates directly with the number of participants who underwent the respective treatment. Furthermore, the thickness of the interconnecting lines is indicative of the volume of randomized controlled trials that conducted direct comparisons between the treatment pairs. The treatments are abbreviated as follows: *R* for remote, *I* for in-person, *e* for electronic health, *m* for mobile health, *PA* for physical activity, *D* for dietary, *E* for education, and *B* for behavior.

Figure 2 presents the network evidence for the included studies, focusing on changes in GWG outcomes. Twenty-six arms were directly compared, forming 15 closed loops. Node size represented the total sample size of each intervention, with I-PAE being the smallest (*n* = 19) and I-B the largest (*n* = 2605). The line width between treatments indicated the volume of comparisons, with the direct evidence between placebo and I-B being more substantial than other comparisons.

### Primary outcome

**FIGURE 3:**
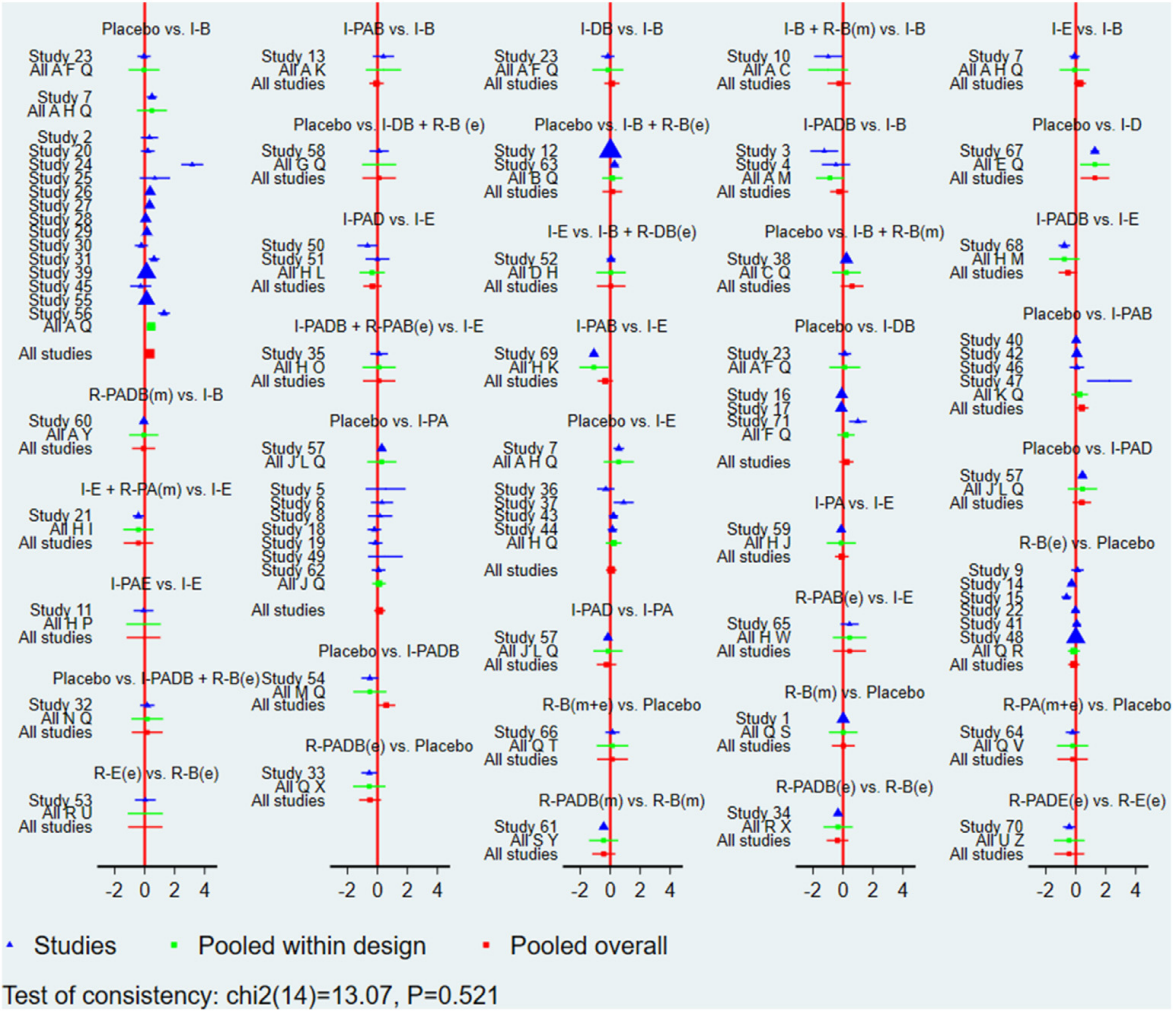
A sophisticated network forest plot delineates the comparative effectiveness of diverse treatment modalities in moderating gestational weight gain outcomes. This plot encapsulates an array of treatment strategies, categorized as follows: A, I-B; B, I-B+R-B(e); C, I-B+RB(m); D, I-B+RDB(e); E, I-D; F, I-DB; G, I-DB+R-B(e); H, I-E; I, I-E+R-PA(m); J, I-PA; K, I-PAB; L, I-PAD; M, I-PADB; N, I-PADB+R-B(e); O, I-PADB+R-PAB(e); P, I-PAE; Q, Placebo; R, R-B(e); S, R-B(m); T, R-B(m+e); U, R-E(e); V, R-PA(m+e); W, R-PAB(e); X, R-PADB(e); Y, R-PADB(m); Z, R-PADE(e). The treatments are abbreviated as follows: *R* for remote, *I* for in-person, *e* for electronic health, *m* for mobile health, *PA* for physical activity, *D* for dietary, *E* for education, and *B* for behavior.

**FIGURE 4:**
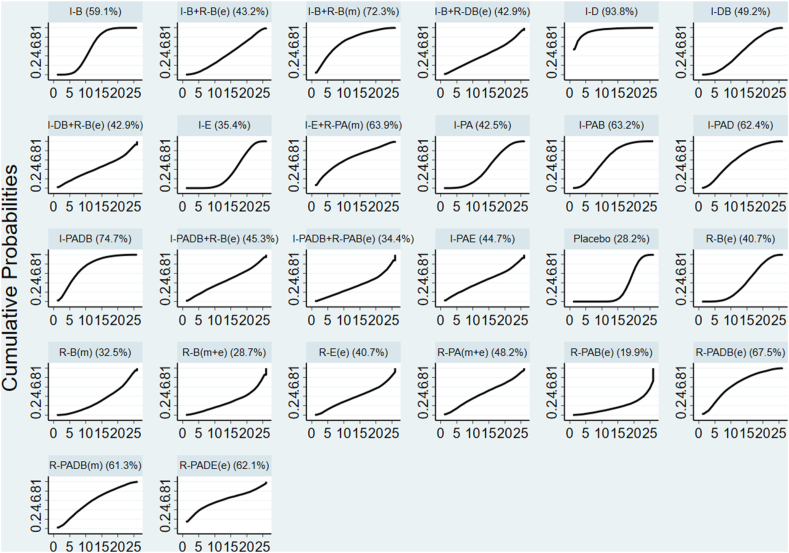
The surface under cumulative ranking (SUCRA) plot constructed based on altered gestational weight gain results. The treatments are abbreviated as follows: *R* for remote, *I* for in-person, *e* for electronic health, *m* for mobile health, *PA* for physical activity, *D* for dietary, *E* for education, and *B* for behavior.

**FIGURE 5:**
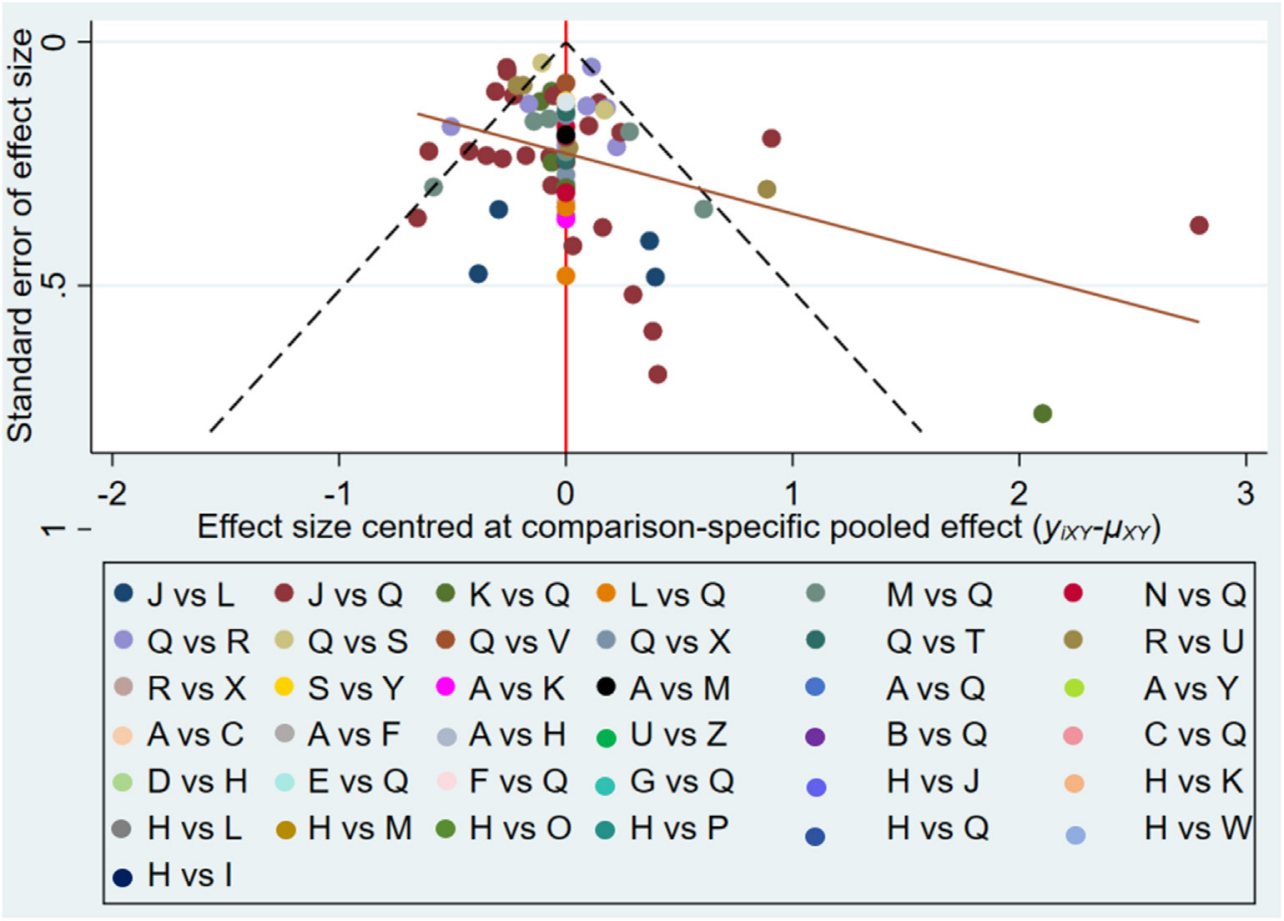
A funnel plot constructed to assess the altered acclimate in gestational weight gain. This plot includes a comprehensive list of treatment combinations, represented by alphabetic designations (A–Z). A, I-B; B, I-B+R-B(e); C, I-B+RB(m); D, I-B+RDB(e); E, I-D; F, I-DB; G, I-DB+R-B(e); H, I-E; I, I-E+R-PA(m); J, I-PA; K, I-PAB; L, I-PAD; M, I-PADB; N, I-PADB+R-B(e); O, I-PADB+R-PAB(e); P, I-PAE; Q, Placebo; R, R-B(e); S, R-B(m); T, R-B(m+e); U, R-E(e); V, R-PA(m+e); W, R-PAB(e); X, R-PADB(e); Y, R-PADB(m); Z, R-PADE(e). The treatments are abbreviated as follows: *R* for remote, *I* for in-person, *e* for electronic health, *m* for mobile health, *PA* for physical activity, *D* for dietary, *E* for education, and *B* for behavior.

**FIGURE 6:**
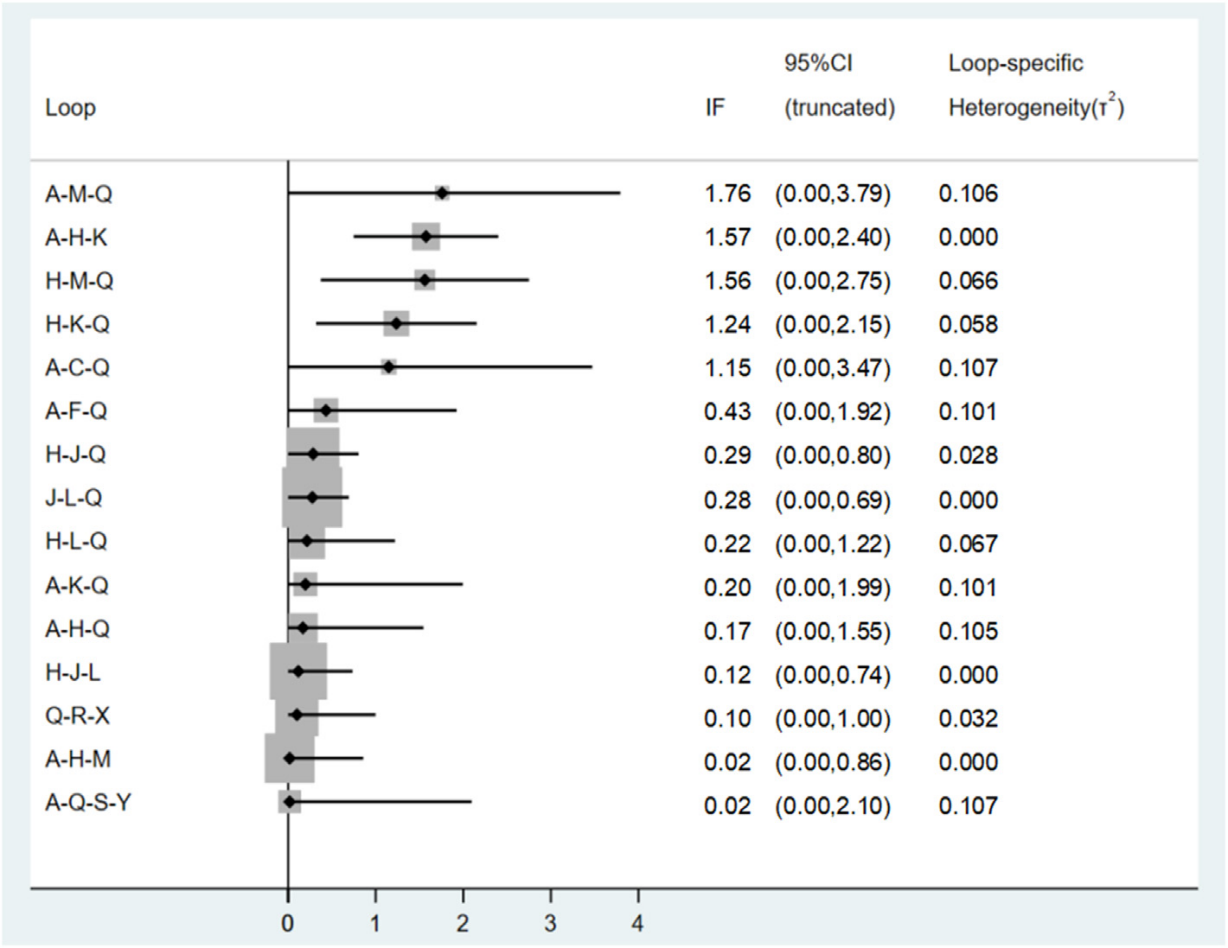
A loop inconsistency plot based on the altered gestational weight gain outcomes. This graph shows the full spectrum of treatment combinations using the standard abbreviations for treatments as outlined: A, I-B; B, I-B+R-B(e); C, I-B + RB(m); D, I-B+RDB(e); E, I-D; F, I-DB; G, I-DB+R-B (e); H, I-E; I, I-E+R-PA(m); J, I-PA; K, I-PAB; L, I-PAD; M, I-PADB; N, I-PADB+R-B(e); O, I-PADB+R-PAB(e); P, I-PAE; Q, Placebo; R, R-B(e); S, R-B(m); T, R-B(m+e); U, R-E(e); V, R-PA(m+e); W, R-PAB(e); X, R-PADB(e); Y, R-PADB(m); Z, R-PADE(e). The treatments are abbreviated as follows: *R* for remote, *I* for in-person, *e* for electronic health, *m* for mobile health, *PA* for physical activity, *D* for dietary, *E* for education, and *B* for behavior. CI, confidence interval; IF, inconsistency factor.

Global inconsistency was low (*I*^2^ = 0%, *P* = 0.521) for GWG changes, as shown in Figure 3. Local inconsistencies were assessed using the node-splitting method. Except for the I-PA compared with placebo comparison (*P* = 0.012), no significant differences were observed in the direct or indirect evidence among treatments (Supplemental Table 5). The interventions I-D (MD: −1.27; 95% CI: −2.23, −0.32), I-PADB (MD: −0.60; 95% CI: −1.19, −0.00), and I-B (MD: −0.34; 95% CI: −0.57, −0.10) demonstrated significant effectiveness over placebo in terms of GWG outcomes. I-D showed markedly higher effectiveness than I-PA, R-B(e), I-E, R-B(m+e), and R-PAB(e) (Supplemental Table 6). No significant discrepancies were noted among the remaining treatments (Supplemental Table 6). The cumulative SUCRA scores rated the intervention effects, with I-D having the highest probability (93.8%) of effectiveness in controlling GWG, followed by I-PADB (74.7%), and then I-B+R-B(m) (72.3%). The intervention R-PAB(e) exhibited the lowest SUCRA score (19.9%) (Figure 4). The comparison-adjusted funnel plot (Figure 5) showed no evidence of publication bias. A loop inconsistency examination of the result indicators suggested the unlikely presence of loop inconsistency (Figure 6).

## Discussion

The present NMA holds the distinction of being the inaugural study to leverage extant data in examining a plethora of treatment modalities for pregnancies complicated by overweight or obesity. An exhaustive analysis was conducted, encompassing 60 RCTs that assessed 26 distinct treatment approaches. These trials collectively involved 16,615 participants. The findings of this evaluation suggest that the I-D treatment manifested a statistically significant superiority in managing GWG in overweight and obese pregnant women, relative to other treatment options. This was followed by I-PADB and I-B+R-B(m). However, the inference that I-D is the preeminent strategy for weight reduction necessitates a prudent interpretation, especially considering the predominance of studies with limited direct and quality evidence.

GWG is a critical and normal physiological process, with its determinants serving as key indirect indicators of both maternal and fetal health. The significance of GWG has been increasingly acknowledged in recent years, a trend accelerated by a surge in related research and the release of GWG guidelines by the IOM [25]. In the review of the 60 included studies on GWG, a significant number (96.6%) adhered to the IOM guidelines as a basis for participant selection and the assessment of experimental outcomes. These guidelines provide a structured framework to evaluate the appropriateness of weight gain during pregnancy based on the prepregnancy BMI of the participants, ensuring that the studies are comparable and their findings applicable to broader public health strategies.

The mode of administration, it appears, exerts a considerable impact on intervention accessibility, quality, and efficacy. This study notes a transformative trend in intervention delivery, adapting to evolving circumstances. It appears that there were few relevant studies conducted between 2002 and 2009. In these studies, FTF intervention dominated the approach to managing GWG in overweight or obese pregnant women. The advent of the hybrid eHealth/FTF mode in 2010, precipitated by technological advances, and the inception of an independent eHealth mode in 2014 [35] signifies a paradigm shift. The mHealth mode, emerging in 2016 after the development of eHealth, has seen a modest decline in the total number of related studies in contrast to the preceding 5 y. Nonetheless, a surge in studies employing the mHealth mode was observed in 2020, potentially as a response to the exigency of maintaining social distancing during the COVID-19 pandemic [36,37]. Despite the convenience and cost-effectiveness of the remote strategy [36], the research indicates a predominance of FTF modes in terms of the volume of existing studies. The unique attributes of pregnancy necessitate FTF sessions for consistent and direct monitoring of weight changes and alterations in diet, PA, and behavior. Such sessions afford instantaneous feedback, aiding pregnant women in implementing crucial adjustments to their diet and PA routines. The primary implementation of remote monitoring, predominantly conducted in isolation, may adversely affect participant motivation and feasibility, especially compared to group settings [38].

In evaluating the efficacy of blended, FTF, and remote delivery modes, a variation was discerned in the effectiveness of different intervention strategies employing these modes for GWG in overweight and obese pregnancies. For instance, compared to R-PADB(e) and R-PADB(m), I-PADB has a superior SUCRA rating; likewise, I-PAB ranks higher than R-PAB(e). However, the efficacy ranking of R-PA(m+e) supersedes that of I-PA; R-E(e) ranks higher than I-E; and the blended administration mode in behavioral intervention outperforms the singular FTF and remote modes. Given these outcomes and the insufficiency of comprehensive data across all intervention approaches in remote, FTF, and hybrid modes, it remains inconclusive to ascertain the most effective among the 3. The efficacy of R-PA and R-E(e) exceeds that of I-PA and I-E, while the effectiveness of I-PAB and I-PADB surpasses R-PAB(e), R-PADB(e), and R-PADB(m). The feasibility of administering a single intervention strategy using a remote mode is acknowledged, yet implementing multiple intervention strategies can be complex and may necessitate careful coordination of various resources [39]. The observed differences in the effects of remote interventions could be attributed to their utilization of distinct platforms across varying time periods. Additionally, the quality of contact, which has not received adequate attention from existing research, may play a significant role in shaping the outcomes of eHealth and mHealth initiatives. Future research endeavors should prioritize exploring the nuances of different eHealth platform usages and their impact over time. This may contribute to a deeper understanding of how eHealth and mHealth interventions can be optimized to achieve the desired health outcomes effectively. The existing protocols in the remote mode may not be conducive to effective management and control of intricate intervention strategies. This necessitates future improvements in remote management and the integration of diverse online resource platforms, taking into account cultural, regional, and economic factors.

A comprehensive and personalized delivery method, integrating multiple intervention strategies, is commonly adopted for managing prenatal weight gain in overweight or obese pregnancies. This study corroborates this approach, noting that the highest number of RCTs, totaling 28, employed multiple intervention modes. However, a singular I-D intervention attained the highest SUCRA score and exhibited a significant impact on reducing gestational weight compared to other modalities. Prior research indicates that dietary management tends to be more efficacious than exercise, lifestyle behavior modification, and education in the short term. However, all these strategies are effective in reducing prenatal weight gain in obese pregnancies [[9], [10], [11],^40^,^41^]. Dietary management is generally more practical and accessible than vigorous exercise, particularly for pregnant women facing physical limitations or discomfort [40]. Exercise interventions during pregnancy must adhere to specific guidelines regarding intensity and duration and usually are conducted during the second trimester in the context of high-risk pregnancies or certain medical conditions [42]. However, dietary intervention allows for early and targeted management to prevent or mitigate many pregnancy complications. Dietary intervention directly influences GWG more than behavior and education intervention; it allows for more precise regulation of calorie intake and can be highly individualized to cater to the specific needs, preferences, and cultural considerations of pregnant women [43]. For instance, in the ID intervention protocol, the experimental group adhered to a balanced nutritional plan ranging from 18 to 24 kcal/kg, comprising 40% carbohydrates, 30% protein, and 30% fat [44]. Additionally, participants in this group maintained daily dietary records and underwent personalized FTF instructional sessions. In contrast, the multiple intervention strategies were utilized in a simple way, focusing solely on limiting sugar, fat, or calorie intake, which may be a factor affecting the effectiveness of the intervention.

Nonetheless, this does not imply that dietary regulation alone suffices for weight reduction during pregnancy for overweight or obese women. Wadden et al. [40] observed that individuals engaging in regular high-intensity PA significantly retained the weight loss outcomes achieved 3 y prior, in contrast to those maintaining low-intensity PA who experienced weight regain. In other words, individuals not participating in regular exercise are more likely to regain weight after rapid weight loss as opposed to those engaging in regular exercise. Regular PA participation may assist in maintaining weight over an extended period of time [40]. Although dietary restriction has a greater impact on weight than exercise alone, it is insufficient for long-term weight and shape maintenance. Therefore, to optimize weight reduction and minimize the likelihood of regaining lost weight, a combination of exercise and a healthy diet is recommended [40].

Weight management programs frequently include dietary counseling, structured PA routines, and behavioral techniques to address lifestyle factors contributing to weight gain [45]. However, the efficacy of these approaches may vary based on individual preferences and readiness for change. This study indicates that a single dietary intervention for weight control in obese pregnant women surpasses pure exercise, lifestyle adjustments, and education interventions in terms of effectiveness. It also outperforms multiple interventions when compared to a placebo (I-D, MD: −1.27; 95% CI: −2.23, −0.32; I-PADB, MD: −0.60; 95% CI: −1.19, −0.00; and I-B, MD: −0.34; 95% CI: −0.57, −0.10). Despite the limited impact of these interventions on pregnancy weight reduction and the absence of significant differences in some studies compared to control groups or IOM recommendations, their clinical relevance should not be overlooked. This may be related to participants who might have already tipped the scales beyond the normal range recommended by the WHO before pregnancy or the time of initial intervention. Such prior weight gains could potentially erode the impact of the intervention, as the window for managing GWG narrows with each advancing stage of pregnancy. Given the methodological heterogeneity of the studies examined, we refrained from addressing the extent to which the timing of intervention initiation during pregnancy influences the efficacy of appropriate GWG. However, this question remains critical and warrants further exploration in subsequent systematic reviews and experimental studies. Although the overall weight change may be minimal, a substantial number of expectant mothers attain weight gain that aligns with the IOM recommendations postintervention. Additionally, the efficacy of these interventions extends beyond mere weight metrics; they demonstrate potential in lowering the occurrence of adverse obesity-related outcomes (gestational diabetes, hypertension, and birth outcomes) both during gestation and postpartum, consequently fostering improved maternal and neonatal health trajectories over the long term [46]. Furthermore, this does not entirely negate the efficacy of multiple intervention strategies. Despite I-D achieving the highest SUCRA rating score of 93.8%, 8 of the top 10 strategies comprise multiple interventions. The effectiveness of these treatments depends on tailoring them to individual needs, providing behavioral support, and meeting the specific requirements of the target group. Owing to the complexity involved in executing multiple interventions, careful coordination is required [47,48]. The studies included in our analysis employed diverse intervention strategies, differing in terms of protocol, including dietary limitations, intensity, and type of PA. Furthermore, the articles exhibit varying levels of bias, particularly in blinding participants and personnel. A growing number of researchers are emphasizing the distinctive complexities associated with the RCT methodology when applied to exercise interventions, which present more substantial challenges than RCTs evaluating pharmacological treatments. In exercise-based RCTs, participants engage in specific exercise programs rather than receiving pharmaceutical interventions. This often necessitates multiple visits to a gymnasium where they perform designated physical activities over a set period. Consequently, it is impractical to maintain blinding in these groups as to whether they are receiving the active intervention or a placebo. Researchers [49] observed that behavioral interventions, including those involving exercise, typically depend on direct interactions between participants and health or exercise professionals. This dynamic complicates the blinding process, as both the participants and the facilitators are inherently aware of the intervention’s nature. Thus, the standard blinding protocol, deemed the gold standard in clinical drug trials, is less applicable and clear-cut in RCTs investigating behavioral interventions [50]. This shift necessitates a re-evaluation of what constitutes methodological rigor in the context of RCTs focused on behavioral and exercise interventions. Although complete double-blinding in pregnancy interventions may not always be feasible, employing a combination of careful design, coupled with the clear segmentation of personnel responsibilities and rigorous adherence to protocols, may maximize the extent of blinding [51]. This, in turn, may enhance the credibility and scientific value of the research findings.

### Strengths and limitations

To the authors’ knowledge, this study represents the first endeavor to assess the effects of remote, FTF, or hybrid administration modalities in conjunction with exercise, nutritional, behavior modification, and education treatments for managing GWG in overweight and obese pregnancies. The utilization of Bayesian NMA, a statistical methodology facilitating the integration of both direct and indirect data by concurrently evaluating multiple treatments and ascertaining their rankings, even without direct comparative evidence, is a pivotal aspect of this study. Obesity during pregnancy is linked to an elevated risk of complications for both the mother and the unborn child. The adoption of effective prevention, intervention, and management strategies can significantly influence the health outcomes of expectant mothers and their infants. Evidence-based strategies provide clinicians and service providers with pragmatic, reliable, and acceptable options for choosing among diverse interventions. However, it is imperative to consider the limitations of this investigation while interpreting the findings. First, the study did not categorize PA intensity or types, which may influence PA efficacy, due to limitations of these data in the studies analyzed. Second, a comparative analysis of the efficacy of remote, FTF, and hybrid modes for each intervention approach was not conducted owing to the dearth of sufficient data on different delivery modes in the current literature. Third, the subjectivity inherent in the results of the ROB assessment must be acknowledged, despite the verification of data consistency by 2 researchers. Fourth, none of the main outcomes from the included studies examined the participants’ acceptability and perceptions of the intervention protocols. Consequently, this information was neither collected nor analyzed in our study. We suggest that future research endeavors include these dimensions to enhance the effectiveness of interventions from the participants’ perspectives. Finally, the complexity inherent in the Bayesian method means that multiple treatments might be challenging to interpret. For instance, the individual diet intervention appears most efficacious compared to multiple treatments, one PA, or behavioral interventions based on an evaluation encompassing a variety of outcomes. This observation may be attributed to the quality of the included studies and the recognition that pregnancy, as a distinct life stage, is more susceptible to various confounding factors that may introduce biases into our initial findings, extending beyond the realm of several interventions.

## Conclusions

We found that the I-D intervention is most effective in managing GWG in obese pregnancies based on our comprehensive analysis, which examined a substantial number of relevant studies. This was closely followed by the I-PADB intervention and the combined I-B+R-B(m) approach. However, we recommend exercising caution when drawing definitive conclusions from these results. The assertion that I-D stands as the optimal treatment among those evaluated should be approached with prudence, primarily due to the low quality of the evidence base and the absence of direct comparative evidence. There exists a pressing need for an evidence-based foundation for intervention strategies targeting overweight and obese pregnancies. Future studies of high-quality are essential to explore and incorporate tailored intervention approaches and administration modes. These studies should aim to fill the current gaps in evidence by providing more robust and direct comparisons. This will enable us to determine the most effective strategies for managing GWG in this demographic. Such research endeavors will guide clinical practice and ensure optimal outcomes for both mothers and their unborn children.

## Author contributions

The authors’ responsibilities were as follows –; HY, ML, SY, GQ: conducted the database search, screened and extracted data for the meta-analysis, prepared extracted data for the procedures; HY, GQ: performed statistical analysis, interpretation of data, and drafted the initial manuscript; HY, GQ: contributed to the discussion and editing; HY, ZO, AS: supervised data collection and critically edited the final manuscript; all authors: agree to be accountable for all aspects of the work; and all authors: read and approved the final manuscript.

## Conflict of interest

The authors report no conflicts of interest.

## Funding

This research was funded in part by the National Science Centre, Poland (No. DEC-2021/05/X/NZ7/01814) within the “HIIT Mama” project: The Effect of Pre- and Postnatal High Intensity Interval Training and Moderate Intensity Continuous Training on Biological, Functional and Psychological Markers of Pregnancy Disorders and Non-communicable Diseases in Mothers and Offsprings (NCT05009433).

## Data availability

Data described in the manuscript, code book, and analytic code will be made available upon request from the corresponding author (HY).

## Declaration of AI and AI-Assisted Technologies in the Writing Process

During the preparation of this work, the authors used Wordtune in order to fix grammatical errors and enhance understanding of context and meaning. After using this tool/service, the authors reviewed and edited the content as needed and take full responsibility for the content of the publication.
