# Supplementary material for: A Systematic Review and Bayesian Network Meta-Analysis Comparing In-Person, Remote, and Blended Interventions in Physical Activity, Diet, Education, and Behavioral Modification on Gestational Weight Gain among Overweight or Obese Pregnant Individuals
Source: Adv Nutr. 2024 Jun 13;15(7):100253. doi: 10.1016/j.advnut.2024.100253 (PMC11267029; doi:10.1016/j.advnut.2024.100253)
Supplement: Multimedia component 1 [file mmc1.docx]

A Systematic Review and Bayesian Network Meta-Analysis Comparing In-Person, Remote, and Blended Interventions in Physical Activity, Diet, Education, and Behavioral Modification on Gestational Weight Gain Among Overweight or Obese Pregnant Individuals

Hongli Yu

**Supplementary Table 2** Search strategy for **Medline (via PubMed), EBSCHost, Embase and Web of Science** Core Collection databases.

Search strategy for **Medline (via PubMed)** (inception-30 June 2023).

| **Category** | **Search terms** |
| --- | --- |
| Population | **#1** pregnan* OR pregnant wom?n OR antenatal OR prenatal OR “prenatal period” OR “expectant mothers” OR “prenatal care” [All Fields]  1,209,624  **#2** obesity OR overweight OR obes* OR adipos* OR “body mass index” OR bmi OR waist OR “body fat” OR “skin fold” OR skinfold [All Fields] 913,790  **#3 #1** AND **#2** 53,588 |
| AND  Intervention | **#4** diet* OR dietary OR nutrition OR “dietary service*” OR “diet service*” OR **“d**iet therap*” OR “restrictive diet therap*” OR “restriction diet therap*” OR “diet restriction*” OR “detary restriction*” OR “diet modification*” OR “dietary modification*” OR “diet counseling” OR “dietary counseling” OR “diet consultation” OR “dietary consultation” OR “dietary management” OR “diet intervention*” OR “dietary intervention*” OR “nutritional consultation” OR “nutritional counseling” [All Fields] 1,533,139  **#5** lifestyle* OR “lifestyle intervention*” OR “life style” OR “lifestyle  management*” [All Fields] 188,646  **#6** exercise OR "physical activity" OR "physical exertion" OR swim* OR gym* OR walk* OR danc* OR jog* OR run* OR cycl* OR bicycl* OR hiking OR "tai ji" OR tai-ji OR "tai chi" OR yoga OR qigong OR "qi gong" OR sport* OR "physical training" OR "strength training" OR "weight training" OR "resistance training" OR "balance training" OR "aerobic training" OR "anaerobic training" OR "endurance training" OR "muscle training" OR exergame OR "active video game" OR wii OR kinect OR pilates OR feldenkrais OR "motor activity" OR "cardiac rehabilitation" [All Fields] 3,034,815  **#7** education OR education OR counselling OR consultation OR booklet OR advice [All Fields] 2,940,196  **#8 #4** OR **#5** OR **#6** OR **#7** 6,903,724 |
| AND  Outcome | **#9** “gestational weight gain” OR “pregnancy weight gain” OR “maternal weight gain” OR “postpartum weight retention” OR “weight control” OR “weight change” OR “weight gain” OR weight OR “body mass” [All Fields] 2,068,150 |
| AND  Study Design | **#10** random* OR rct OR “clinical trial” OR “intervention study” OR “interventional study” [All Fields] 2,185,923 |
| Result | **#11 #3 AND #8 AND #9 AND #10** 3,515 |

Search strategy for **Embase** (inception-30 June 2023).

| **Category** | **Search terms** |
| --- | --- |
| Population | **#1** 'pregnan*'/exp OR 'pregnant wom?n'/exp OR 'antenatal'/exp OR 'prenatal'/exp OR 'prenatal period'/exp OR 'expectant mothers'/exp OR 'prenatal care'/exp 1,326,405  **#2** 'obesity'/exp OR 'overweight'/exp OR 'obes*'/exp OR 'adipos*'/exp OR 'body mass index'/exp OR 'bmi'/exp OR 'waist'/exp OR 'body fat'/exp OR 'skin fold'/exp OR 'skinfold'/exp 1,417,929  **#3 #1** AND **#2** 84,057 |
| AND  Intervention | **#4** 'diet*'/exp OR 'dietary'/exp OR 'nutrition'/exp OR 'dietary Service*'/exp OR 'diet service*'/exp OR 'diet therap*'/exp OR 'restrictive diet therap*'/exp OR 'restriction diet therap*'/exp OR 'diet restriction*'/exp OR 'dietary restriction*'/exp OR 'diet modification*'/exp OR 'dietary modification*'/exp OR 'diet counseling'/exp OR 'dietary counseling'/exp OR 'diet consultation'/exp OR 'dietary consultation'/exp OR 'dietary management'/exp OR 'diet intervention*'/exp OR 'dietary intervention*'/exp OR 'nutritional consultation'/exp OR 'nutritional counseling'/exp 3,320,350  **#5** 'lifestyle*'/exp OR "lifestyle intervention*'/exp OR "life style'/exp OR "lifestyle  Management*'/exp 301,505  **#6** 'exercise'/exp OR 'physical activity'/exp OR 'physical exertion'/exp OR 'swim*'/exp OR 'gym*'/exp OR 'walk*'/exp OR 'danc*'/exp OR 'jog*'/exp OR 'run*'/exp OR 'cycl*'/exp OR 'bicycl*'/exp OR 'hiking'/exp OR 'tai ji'/exp OR 'tai-ji'/exp OR 'tai chi'/exp OR 'yoga'/exp OR 'qigong'/exp OR 'qi gong'/exp OR 'sport*'/exp OR 'physical training'/exp OR 'strength training'/exp OR 'weight training'/exp OR 'resistance training'/exp OR 'balance training'/exp OR 'aerobic training'/exp OR 'anaerobic training'/exp OR 'endurance training'/exp OR 'muscle training'/exp OR 'exergame'/exp OR 'active video game'/exp OR 'wii'/exp OR 'kinect'/exp OR 'pilates'/exp OR 'feldenkrais'/exp OR 'motor activity'/exp OR 'cardiac rehabilitation'/exp 5,108,831  **#7** 'education'/exp OR 'education session'/exp OR 'counselling'/exp OR 'consultation'/exp OR 'booklet'/exp OR 'advice'/exp 2,126,524  **#8 #4** OR **#5** OR **#6** OR **#7** 9,660,488 |
| AND  Outcome | **#9** 'gestational weight gain'/exp OR 'pregnancy weight gain'/exp OR 'maternal weight gain'/exp OR 'postpartum weight retention'/exp OR 'weight control'/exp OR 'weight change'/exp OR 'weight gain'/exp OR 'weight'/exp OR 'body mass'/exp 1,705,919 |
| AND  Study Design | **#10** 'random*'/exp OR 'RCT'/exp OR 'clinical trial*'/exp OR 'intervention study'/exp OR 'interventional study'/exp 2,705,466 |
| Result | **#11 #3 AND #8 AND #9 AND #10** 5,350 |

Search strategy for **EBSCHost** (inception-30 June 2023).

| **Category** | **Search terms** |
| --- | --- |
| Population | **#1** TX pregnan* OR pregnant wom?n OR antenatal OR prenatal OR prenatal period OR expectant mothers OR prenatal care 3,836,503  **#2** TX obesity OR overweight OR obes* OR adipos* OR "body mass index" OR bmi OR waist OR body fat OR skin fold OR skinfold 2,805,724  **#3 #1** AND **#2** 273,404 |
| AND  Intervention | **#4** TX diet* OR dietary OR nutrition OR "dietary Service*" OR "diet Service*" OR "diet therap*" OR "restrictive diet therap*" OR "restriction diet therap*" OR "diet restriction*" OR "dietary restriction*" OR "diet modification*" OR "dietary modification*" OR "diet counseling" OR "dietary counseling" OR "diet consultation" OR "dietary consultation" OR"dietary management" OR "diet intervention*" OR "dietary intervention*" OR "nutritional consultation" OR "nutritional counseling" 7,631,259  **#5** TX lifestyle* OR "lifestyle intervention*" OR "life style" OR "lifestyle  Management*" 4,055,223  **#6** TX exercise OR "physical activity" OR "physical exertion" OR swim* OR gym* OR walk* OR danc* OR jog* OR run* OR cycl* OR bicycl* OR hiking OR "tai ji" OR tai-ji OR "tai chi" OR yoga OR qigong OR "qi gong" OR sport* OR "physical training" OR "strength training" OR "weight training" OR "resistance training" OR "balance training" OR "aerobic training" OR "anaerobic training" OR "endurance training" OR "muscle training" OR exergame OR "active video game" OR wii OR kinect OR pilates OR feldenkrais OR "motor activity" OR "cardiac rehabilitation" 40,333,222  **#7** TX education OR education OR counselling OR consultation OR booklet OR advice 23,462,427  **#8 #4** OR **#5** OR **#6** OR **#7** 44,317,932 |
| AND  Outcome | **#9** TX "gestational weight gain" OR "pregnancy weight gain" OR "maternal weight gain" OR "postpartum weight retention" OR "weight control" OR "weight change" OR "weight gain" OR weight OR "body mass" 4,228,081 |
| AND  Study Design | **#10** TX random* OR RCT OR "clinical trial" OR "intervention study" OR "interventional study" 10,026,797 |
| Result | **#11 #3 AND #8 AND #9 AND #10** 32,347 |

Search strategy for **Web of Science Core Collection databases** (inception-30 June 2023).

| **Category** | **Search terms** |
| --- | --- |
| Population | **#1** ALL=(Pregnan* OR pregnant wom?n OR antenatal OR prenatal OR prenatal period OR expectant mothers OR prenatal care) 771,974  **#2** ALL=(obesity OR Overweight OR obes* OR adipos* OR "body mass index" OR bmi OR waist OR body fat OR skin fold OR skinfold) 1,052,487  **#3 #1** AND **#2** 50,677 |
| AND  Intervention | **#4** ALL=(diet* OR dietary OR nutrition OR "dietary Service*" OR "diet Service*" OR "Diet Therap*" OR "Restrictive Diet Therap*" OR "Restriction Diet Therap*" OR "Diet Restriction*" OR"Dietary Restriction*" OR "Diet Modification*" OR "Dietary Modification*" OR "diet counseling" OR "dietary counseling" OR "diet consultation" OR "dietary consultation" OR"dietary management" OR "diet intervention*" OR "dietary intervention*" OR "nutritional consultation" OR "nutritional counseling") 2,119,224  **#5** ALL=(lifestyle* OR "lifestyle intervention*" OR "life style" OR "lifestyle  Management*") 218,128  **#6** ALL=(exercise OR "physical activity" OR "physical exertion" OR swim* OR gym* OR walk* OR danc* OR jog* OR run* OR cycl* OR bicycl* OR hiking OR "tai ji" OR tai-ji OR "tai chi" OR yoga OR qigong OR "qi gong" OR sport* OR "physical training" OR "strength training" OR "weight training" OR "resistance training" OR "balance training" OR "aerobic training" OR "anaerobic training" OR "endurance training" OR "muscle training" OR exergame OR "active video game" OR wii OR kinect OR pilates OR feldenkrais OR "motor activity" OR "cardiac rehabilitation") 6,919,072  **#7** ALL=(education OR education OR counselling OR consultation OR booklet OR advice) 6,697,470  **#8 #4** OR **#5** OR **#6** OR **#7** [13,878,647](https://www.webofscience.com/wos/woscc/summary/6ca94858-ac76-4493-84a0-5e5f1112b7aa-c5434a7f/relevance/1) |
| AND  Outcome | **#9** ALL=("Gestational weight gain" OR "Pregnancy Weight Gain" OR "Maternal Weight Gain" OR "Postpartum Weight Retention" OR "weight control" OR "weight change" OR "weight gain" OR weight OR "body mass") [2,491,893](https://www.webofscience.com/wos/woscc/summary/f6c6540a-6be3-41bb-8845-9e45e6acaa0b-c543549b/relevance/1) |
| AND  Study Design | **#10** ALL=(random* OR RCT OR "clinical trial" OR "intervention study" OR "interventional study") 2,612,059 |
| Result | **#11 #3 AND #8 AND #9 AND #10** 2,191 |

**Supplementary Table 3** Baseline characteristics of the 60 included trials

| Author  (year) | Participants characteristics | | | | | Intervention characteristics | | | | Control group | Main outcome |
| --- | --- | --- | --- | --- | --- | --- | --- | --- | --- | --- | --- |
|  | Ethnicity/Country  (%) | Age  (M±SD) or (median) | Gestation  week  (M±SD) | Sample size  (N) | Participant type | Delivery  setting | Behaviour targets  (type) | Modality and intensity | Intervention length |  |  |
| Ainscough  (2020) | White (I: 94.8%, C: 91.1%), others (I: 5.2%, C: 8.9%) | I: 32.8 ± 4.6  C: 32.2 ± 4.2 | T: 15.6 | I:271  C:283 | OB/OW | Mobile application | B (PA and D) | Daily application usage | From 16 to 34 gestation weeks | Routine prenatal care | GWG  PAL  Energy and macro-nutrient |
| Althuizen (2013) | Netherlands  (I: 92%, C: 95%), other (I: 8%, C: 5%) | I: 29.2 ± 3.8  C: 30.4 ± 4.0 | ≥ 15 weeks | I:25  C:22 | OB/OW | At clinic | B (PA and D) | 5 individual sessions  (15mins/session, the first session 30 min) | From 15 gestation weeks to term | Routine prenatal care | GWG |
| Asbee (2009) | African American (I: 26.3%, C: 21.4%),  Asian (I: 5.3%, C: 2.4%), White (I: 8.8%, C: 19%,  Hispanic (I: 57.9%, C: 54.8%), other (I: 1.8%, C: 2.4%) | I: 26.7 ± 6.0  C: 26.4 ± 5.0 | I:13.7 ± 3.6  C: 13.6 ± 3.2 | I:57  C:43 | OB/OW | At clinic | PA (moderate),  D (40% carbohydrate; 30% protein; 30% fat)  B (PA and D) | 48 to 80 sessions (NR)  4 individual sessions at  prenatal care (NR) | 16 weeks | B (PA and D) | BMI change  Body weight  GWG |
| Barakat  (2011) | Caucasian (100%) | I: 32 ± 4.0  C: 31 ± 3.0 | ≥ 6 weeks | I:40  C:43 | OB/OW | At non-clinic setting by exercise specialists | PA (light to moderate) | 85 training sessions (35-45mins/session) | From 6 gestation weeks to term | Routine prenatal care | 50g MGS  GDM  Body weight  BMI  GWG |
| Bogaerts (2013) | Belgian/Dutch (I^A^: 81.6%, I^B^: 81%, C: 77.8%),  Turkish/Moroccan (I^A^: 10.5%, I^B^: 13.8%, C: 14.3%), other (I^A^: 7.9%, I^B^: 5.2%, C: 7.9%) | I^A^: 28.7 ± 4.2  I^B^: 29.6 ± 4.9  C: 28.8 ± 4.5 | ≤ 15 weeks | I^A^: 76  I^B^: 58  C: 63 | OB | At clinic | B (D)  E (D) | 1 individual and 3  group session (in total 1.5 -2 hours) | From 15 gestation weeks to term | Routine prenatal care | BMI  Body weight  GWG  GDM |
| Byrne (2011) | NR | I: 31.1 ± 3.0  C: 31.6 ± 3.1 | ≤ 12 weeks | I: 11  C: 12 | OB | At clinic | PA (walking) | Monthly individual,  phone call or group  sessions (NR) | From 15 to 30 gestation weeks | Routine prenatal care | Height  Skinfold thicknesses  BMI  GWG  RMR  Walking speed Metabolic cost of walking  Body composition |
| Dahl  (2020) | White (T: 81.6%) | I: 30.2 ± 3.6  C: 29.9 ± 3.1 | I: 15.9 ± 5.4  C: 14.7 ± 4.4 | I: 40  C: 47 | OB/OW | Mobile application | B (PA and D) | Daily behavioral challenge on 5 of the 7 days of the week | 12 weeks | Received materials focused on stress reduction and management | GWG |
| Darvall (2022) | NR | I: 28.4 ± 5.8  C: 30.0 ± 5.0 | Within 10 gestation weeks | I: 10  C: 10 | OB | Mobile application  At home | B (PA and D) | PA (once activity  exceeded 3 metabolic equivalents for ≥10 min) | Up to 32 weeks of gestation | B (healthy eating and  healthy lifestyle) | GWG |
| Dekker Nitert (2015) | NR | I: 33.3 ± 5.6  C: 30.8 ± 4.9 | 12 weeks | I: 16  C: 19 | OB | Location  was not reported | PA (individualized),  E (PA and D guidance) | Monthly individual and group sessions (NR) | From 12 to 36 gestation weeks | E (PA and D guidance) | GWG  PAL  Fasting glucose |
| Dodd  (2014) | Caucasian (I: 90.7%, C: 91.2%) | I: 29.4 ± 5.4  C: 29.6 ± 5.4 | 14.3 weeks | I: 1080  C: 1072 | OB/OW | At clinic | B (PA and D) | 1 individual and 5 phone calls (NR) | From 22 to 36 gestation weeks | Routine prenatal care | Healthy eating index  Micronutrient consumption  Glycaemic load and glycaemic index  PAL  BMI  GWG |
| Downs  (2021) | Caucasian/white (I: 93%, C: 100%),  Asian (I: 7%, C: 0%) | I: 29.7 ± 3.7  C: 29.6 ± 4.5 | I: 10.3 ± 1.6  C: 10.1 ± 1.7 | I: 15  C: 16 | OB/OW | At clinic, community-based, and Web-based | B (PA and D)  PA (aerobics, swimming, resistance activities) | B (Maximum of 24 weekly modules, 45-60 min/week)  PA (45 min/session) | Up to 38 gestation weeks | B (healthy eating and  healthy lifestyle) | GWG |
| Ferrara  (2020) | Asian (I:22%, C: 20%),  White (I: 32%, C: 33%),  Hispanic (I: 20%, C: 20%),  African American (I: 8%, C: 8%) | I: 32·4 ± 4·1  C: 32·6 ± 4·3 | < 13 weeks | I: 194  C: 194 | OB/OW | At home | B (PA and D) | 13 weekly individuals  sessions (The first and last sessions were in person, 11 were delivered by telephone) | Up to 32 gestation weeks | Routine prenatal care | GWG |
| Garmendia  (2021) | NR | I^A^: 28.4 ± 5.7  I^B^: 27.3 ± 5.9  I^C^: 27.6 ± 5.6  C: 28.1 ± 5.7 | < 15 weeks | I^A^: 250  I^B^: 252  I^C^: 249  C: 251 | OB/OW | At home | D (Group 1: home based dietary counseling plus 800 mg/ day DHA supplementation (n¼250); Group 2: routine counseling plus  800 mg/day DHA (n¼252); Group 3: home-based dietary counseling plus  200 mg/day DHA (n¼249); and Group 4: routine counseling plus 200 mg/day DHA (n¼251))  B (PA and D) | 3 times counseling sessions | Up to 37 gestation weeks | Routine prenatal care | GDM  GWG |
| Garnæs (2016) | NR | I: 31.3 ± 3.8  C: 31.4 ± 4.7 | ≥ 12 weeks | I: 38  C: 36 | OB/OW | At clinic | PA (moderate, endurance and resistance training) | 3 times/week  group sessions (60mins/session) and 1times home exercise per week (50mins/sessions | From 12 - 18 to 34 - 37  gestation weeks | Routine prenatal care | GWG |
| Garnæs  (2018) | NR | I: 31.6 ± 3.6  C: 31.3 ± 4.6 | 16 - 18 weeks | I: 36  C: 34 | OB/OW | At clinic | PA (endurance training and resistance training, home exercise, pelvic  floor muscle exercises) | PA (3 times/week, 35 min endurance training and resistance training; daily home exercise; daily pelvic floor muscle exercises) | From 12–18 gestation weeks until delivery | Routine prenatal care | GWG  BMI  GDM |
| Gesell  (2015) | Hispanic (I: 37%, C: 43%),  Non-Hispanic White 4 (I: 5%, C: 1%),  African–American (I: 6%, C: 3%),  Other 1 (I: 1%, C:3%) | I: 25.8 ± 6.6  C:27.5 ± 5.8 | 10 - 28 weeks | I: 36  C: 38 | OB/OW | In community | B (PA, D, and sleep hygiene) | 12 sessions (90 min/session) | 12 weeks | Routine prenatal care | GWG |
| Gonzalez-Plaza (2022) | Spanish  (I: 51%,  C: 57%),  Foreign  I: 49%,  C: 43%) | I: 32.4 ± 5.4  C: 33.4 ± 4.7 | 12 to 18 weeks | I: 60  C: 53 | OB/OW | Mobile application  At clinic | PA (moderate)  E (D guidance) | PA (10,000 steps a day, 30 minutes per day) | From 12 to 37 gestation weeks | E (healthy eating and  healthy lifestyle guidance) | GWG |
| Graham  (2014) | White (T: 63.8%) Black (T: 24.4%,)  Other (T: 10%),  Hispanic (T: 1.8%) | 18–35 years | NR | I: 164  C: 89 | OB/OW | At home | B (PA and D) | Visit Web site.  Weekly e-mail  reminders to visit the site | During pregnancy | Routine prenatal care | GWG |
| Guelinckx (2010) | Caucasians  (100%) | I^A^: 28.7 ± 4.0  I^B^: 28.0 ± 3.6  C: 29.4 ± 4.4 | I^A^: 10.2 ± 2.6  I^B^: 9.3 ± 2.8  C: 10.2 ± 2.4 | I^A^: 37  I^B^: 42  C: 43 | OB | At clinic | D (limiting  the intake of energy-dense foods),  E (PA and D guidance),  B (PA and D) | 3 sessions (1 hour/session) | From 16 to 37 gestation weeks | Routine prenatal care | Dietary habits  PAL  GWG |
| Hajian  (2020) | NR | I:25.9 ±4.2  C:25.1 ± 3.4 | 16–20 weeks | I:33  C:33 | OW | NR | B (PA and D) | 3 counselling sessions (45 - 60 min/session) | From 16 to 37 gestation weeks | Routine prenatal care | GWG |
| Harden (2014) | Caucasian (T:61.5%), black  (T: 23%), multiracial (T: 7.6%), and unreported (T: 7.6%) | T: 21.9 ± 4.8 | ≤ 21 weeks | I: 8  C: 8 | OB | At clinic | B (PA and D) | 6 sessions (1 hour/session) | From 12 to 36 gestation weeks | NR | GWG |
| Harreiter  (2019) | NR | T^A^: 32.2 ± 5.4  T^B^: 31.7 ± 5.3  T^C^: 31.8 ± 5.2  T^D^: 32.1 ± 5.5 | < 20 weeks | I^A^: 192  I^B^: 170  I^C^: 189  I^D^: 166  C^A^: 193  C^B^: 162  C^C^: 196  C^D^: 166 | OB | At home | B (PA and D) | 5 counselling sessions, 4 telephone calls, and e-mail contacts | Up to 37 gestation weeks | Routine prenatal care | GWG  GDM |
| Harrison (2013) | Australia (I: 44%, C: 41%), Southeast Asia (I: 16%, C: 13%),  Southern/Central Asia (I:43%, C: 38%),  Other (I: 18%, C: 15%) | I: 32.4 ± 4.6  C: 31.7 ± 4.5 | T: 14 ± 0.8 | I: 97  C: 106 | OB/OW | At clinic | B (PA and D) | 4 individuals  session (NR) | From 14 to 28 gestation weeks | A brief, single PA and D guidance | GWG |
| Hawkins (2015) | Hispanics (T:100%) | T:18-40 | 14.9 weeks | I: 32  C: 34 | OB/OW | NR | PA (moderate)  D (saturated fat and dietary fibers)  B (PA and D) | 6 behavioral counselling sessions (NR) and 5 telephone-delivered booster sessions (NR) | 6 months | Routine prenatal care | PAL  GWG |
| Herring (2016) | African American (T: 100%) | I: 25.9 ± 4.9  C: 25.0 ± 5.7 | I:11.5 ± 2.9  C: 13.4 ± 4.1 | I:27  C: 29 | OB/OW | In community | PA (Walk 5,000 steps daily),  D (Limit sugar-sweetened beverages  to 1 cup per day; limit junk and high fat food to no more than  1 per day; stick to 1 plate of food at each meal)  B (PA and D) | Weekly counselling (15-20 min),  daily text messages,  and Facebook | Form 12 gestation weeks to term | Routine prenatal care | Weight  GWG |
| Horn  (2018) | White (I: 56.4%, C: 70.2%),  Black or African American (I: 24.3%, C: 14.2%),  Other (I: 19.3%, C: 15.6%) | I: 33 ± 4  C: 34 ± 4 | I: 13 ± 1  C: 13 ± 1 | I: 139  C: 135 | OB/OW | At home | PA (activity or walking)  D (DASH diet:)  B (PA and D) | PA (engage in >30 min of activity or walking >10,000 steps per day)  3 individual and 6 group counseling sessions | Up to 35 gestation weeks | B (PA and D) | GWG |
| Hui  (2006) | Aboriginal (I: 38%, C: 57%%) | I: 26.2 ± 5.4  C: 26.2 ± 5.7 | < 26 weeks | I: 24  C: 21 | OB/OW | In community | PA (group exercise or  a DVD at home), D (use of a software)  B (PA and D) | Weekly group-session exercises (45 min/session) and home-based exercise (NR) | From 20 to 36 gestation weeks | E (PA and D guidance) | GWG  GDM |
| Jeffries (2009) | NR | T:18-45 | ≤ 14 weeks | I: 45  C: 39 | OB/OW | At home | E (PA and D guidance) | Individual exchange  at prenatal care | From 16 to 34 gestation weeks | Routine prenatal care | BMI  GWG |
| Kennelly  (2018) | Caucasian (I: 93.2%, C: 89.2%),  Non-Caucasian (I: 6.8%, C: 10.8%) | I: 32.8 ± 4.6  C: 32.1 ± 4.2 | 10 - 15 weeks | I: 278  C: 287 | OB/OW | Mobile application  At home | B (PA and D) | Emails every 2 weeks; face-to-face hospital visits twice; daily used smart phone application. | Up to 34 gestation weeks | Routine prenatal care | GWG  GDM |
| Koivusalo (2016) | NR | I: 32.3 ± 4.9  C: 32.6 ± 4.5 | 13.3 weeks | I: 144  C: 125 | OB | In community | B (PA and D)  PA (Moderate) | 3 individual counselling session, 1 group session (2 hours) | From 20 weeks to term | A brief, single PA and D guidance | GWG  GDM |
| Krebs  (2022) | NR | I: 31.3 ± 4.3  C: 31.3 ± 4.4 | I: 9.9 ± 1.9  C: 9.9 ± 2.0 | I: 792  C: 674 | OB/OW | In community | B (PA and D) | six brief counseling  sessions | 6 months | Routine prenatal care | GWG |
| Liu  (2021) | White (I:58%, C:42%),  African American (I:52.4%, C:47.6%) | I: 30.4 ± 5.1  C: 29.1 ± 4.8 | ≤ 16 weeks | I:112  C:105 | OB/OW | At home | B (D and PA) | 6 monthly mailings and 10 weekly podcasts | Up to 32 gestation weeks | Routine prenatal care | GWG |
| Luoto  (2011) | Finland (NR) | I: 29.5 ± 4.8  C: 30.0 ± 4.7 | 8 -12 weeks | I: 216  C: 179 | OW | At clinic | PA (NR)  B (PA and D) | 7 sessions counselling (NR), group session (NR) | From 8 to 37 gestation weeks | A simple dietary and PA counseling | GDM  GWG |
| McCarthy (2016) | Australia (I: 77.4%, C: 74.0%) | I: 31.9 ± 4.6  C: 31.8 ± 4.6 | I:14.7 ± 2.8  C: 14.7 ± 2.6 | I: 159  C: 154 | OB/OW | At clinic | E (D) | 1 individual session (30 min) | Form the first or second antenatal visit to term | Routine prenatal care | GWG  GDM |
| McNitt  (2022) | White (T: 100%) | T: 30.7 ± 3.0 | T:10.0 ± 1.7 | I: 15  C: 16 | OB/OW | NR | B (D and PA) | NR | from 8–12 to 36 gestation weeks | Routine prenatal care | GWG |
| Nascimento (2011) | NR | I: 29.7 ± 6.8  C: 30.9 ± 5.9 | I:14.3 ± 4.5  C: 13.6 ± 3.5 | I: 39  C: 41 | OB/OW | At clinic | PA (Light to moderate),  B (PA and D guidance) | Weekly group sessions (40 min); home exercise counselling (5 times/week) | From 14 gestation weeks to term | Standard nutrition counseling | GWG |
| Olson  (2018) | Non-Hispanic white (I: 64.8%, C: 63.8%),  Non-Hispanic African American (I: 21.1%, C: 23.5%),  Hispanic/Latina (I: 5.1%, C: 7.0%),  Other (I: 9.0%, C: 5.7%) | 18-35 years | ≤ 20 weeks | I: 563  C: 1126 | OB/OW | At home | B (PA and D) | Weekly (NR) | Up to 37 gestation weeks | Routine prenatal care | GWG |
| Ong  (2009) | NR | T: 30 ± 4.0 | 18 weeks | I: 6  C: 6 | OB | At non-clinic setting | PA (moderate, stationary cycling) | Home-based exercise (3 times/week, 50 min/session) | 10 weeks | Routine prenatal care | PAL  GWG  OGTT |
| Petrella (2014) | Caucasian (I: 84.9%, C: 66.7%), Maghreb (I: 12.1%, C: 20%), other (I: 3%, C: 13.3%) | I: 31.5 ± 4.2  C: 32.4 ± 5.9 | 12 weeks | I: 33  C: 28 | OB/OW | At clinic | PA (moderate),  D (OW: 1700 kcal/day, OB:  1800 kcal/day) | 3 times a week (30 min/session) | From 16 to 36 gestation weeks | A simple nutritional booklet | GWG |
| Phelan (2011) | Non-Hispanic white (I: 68.7%, C: 67.5%),  Latina and Hispanic (I: 19.6%, C: 19.6%),  Non-Hispanic African American (I: 7.1%, C: 9.6%),  Other (I: 4.6%, C: 3.3%) | I: 28.6 ± 5.2  C: 28.8 ± 5.2 | I: 13.6 ± 1.8  C: 13.5 ± 1.8 | I: 87  C: 90 | OB/OW | At research facility | B (PA and D)  D (20 kcal/kg) | 3 brief support phone calls and weekly postcards | From 16 gestation weeks to term | Standard nutrition counseling | GWG |
| Pollak  (2014) | White (I: 53%, C: 41%) | I: 29 ± 5  C:32 ± 2 | I: 16 ± 3  C: 17 ± 3 | I: 23  C: 12 | OB/OW | Mobile application | B (PA and D guidance)  PA (walking 10,000 steps) | Text 3 times/week | Up to 36 gestation weeks | Text 3 times/week with general  pregnancy information | GWG |
| Polley  (2002) | Black (T: 39%),  White (T: 61%) | T: 25.5 ± 4.8 | T: 14.5 ± 3.1 | I: 27  C: 22 | OW | At clinic Phone call at home | PA (walking)  D (decreasing high-fat foods)  B (PA and D) | Stepped care approach (NR) | From 20-30 weeks gestation and 6 weeks postpartum | A simple D guidance | GWG  Behavior changes |
| Poston  (2015) | White (I: 63%, C: 63%),  Black (I: 26%, C: 26%),  Asian (I: 6%, C: 6%),  Other (I: 6%, C: 5%) | I: 30·4 ± 5·6 C: 30·5 ± 5·5 | 15 - 18 weeks + 6 days | I: 526  C: 567 | OB | At clinic | B (PA and D) | 8 individual sessions (1 time/week, 1 h/session) | From 15 - 18 weeks + 6 days to 27 - 28 weeks + 6 days of  gestation | Routine prenatal care | GWG |
| Quinlivan (2011) | Asian (I: 13%, C: 26%),  Caucasian (I: 79%, C: 67%),  Other (I: 8%, C: 7%) | I: 28.3 ± 0.6  C: 29.5 ± 0.7 | During prenatal care | I: 63  C: 61 | OB/OW | At clinic | B (D) | 4 individual sessions (NR) | During prenatal care | Routine prenatal care | GWG  GDM  OGTT |
| Redman  (2017) | Black (I^A^: 28%, I^B^: 11%, and C: 35%),  White (I^A^: 61%, I^B^: 65%, and C: 84%),  Other (I^A^: 0%, I^B^: 5%, and C: 11%) | I^A^: 29.2 ± 4.8  I^B^: 29.0 ± 4.2  C: 29.5 ± 5.1 | 10.4 - 13.6 weeks | I^A^: 18  I^B^: 19  C: 17 | OB/OW | Mobile application  At clinic | PA (walking)  D (based on IOM guidance)  E (PA and D guidance) | 18 sessions (NR) | Up to 36 gestation weeks | E (PA and D guidance) | GWG |
| Renault (2014) | Caucasian (IA: 98%, IB: 98% and C: 97%) | I^A^: 31.2 ± 4.4  I^B^: 30.9 ± 4.9  C: 31.3 ± 4.2 | < 16 weeks | I^A^: 130  I^B^: 125  C: 134 | OB | At clinic | PA (11,000 steps daily),  D (Mediterranean-style diet) | Text message, 11 - 13 times phone calls (1 time per 2 weeks) | From 16 gestation weeks to term | Received the usual hospital standard  regimen for obese pregnant women | GWG  PAL |
| Rhodes (2010) | Non-Hispanic white (I: 56%, C: 52%),  Non-Hispanic black (I: 12%, C: 10%),  Hispanic (I: 20%, C: 14%),  Asian (I: 0%, C: 5%),  Non-Hispanic mixed (I: 8%, C: 5%),  Other/unknown (I: 4%, C: 14%) | I: 28.3 ± 0.6  C: 29.5 ± 0.7 | I: 19.8 ± 5.0  C: 19.6 ± 4.3 | I: 22  C: 16 | OB/OW | At clinic | D (low-glycemic load diet),  B (D) | One in-person and weekly phone counseling sessions after 36 weeks (NR),  every 2 - 4 weeks  individual sessions (NR) | Second trimester to 36 gestation weeks | Routine prenatal care | Weight  Blood pressure  Urine analysis for ketones  GWG |
| Ruiz  (2013) | NR | I: 31.6 ± 4.0  C: 31.9 ± 4.0 | 5 - 6 weeks | I: 146  C: 129 | OB/OW | NR | PA (light- to moderate-intensity aerobic and resistance exercises) | 85 training sessions (3 times/week, 50-55 min/session). | From 9 to 38 - 39 gestation weeks | A simple dietary and PA counseling | GWG |
| Sandborg (2021) | Swedish  (T: 100%) | I: 31.4 ± 4.3  C: 31.3 ± 3.8 | I: 13.8 ± 0.6  C: 14.0 ± 0.7 | I:122  C:125 | OB/OW | Mobile application  At home | PA (aerobic and  resistance exercises and training programs)  B (PA and D)  D (diet guidance) | 4 times/week | From 14.9 ± 0.7 to 36.4 ± 0.4 geastation weeks | B (PA and D) | GWG  BMI |
| Sandborg (2022) | Swedish (I: 91.0%, C: 86.4%) | I: 31.5 ± 4.3 C: 31.2 ± 3.8 | I: 13.8 ± 0.6 C:14.0 ± 0.8 | I: 137  C: 134 | OB/OW | Mobile application | PA (application exercise module)  D (application dietary module)  B (D and PA) | Utilized 6 months application | 6 months | Regular midwife visits and an optional  lecture | GWG |
| Santos (2005) | NR | I: 26.0 ± 3.4  C: 28.6 ± 5.9 | I: 17.5 ± 3.3  C: 18.4 ± 3.9 | I: 37  C: 35 | OW | At non-clinic setting | PA (moderate aerobics) | Group exercise sessions (3 times/week, 1hour/session) | From 20 to 32 gestation weeks | Weekly relaxation and focus group discussions | VO_2_  GWG |
| Sartorelli  (2023) | White (I: 30.3%, C: 32.3%),  Black (I: 14.6%, C: 15.8%),  Mulatto (I: 53.0%, C: 53.9%) | I: 27  (median)  C: 27  (median) | I: 11  (median)  C: 11  (median) | I: 97  C: 110 | OW | At home | B (D and PA) | 3 individualized nutritional counselling sessions (30 min/session) | Up to 33 gestation weeks | Routine prenatal care | GWG |
| Seneviratne (2016) | Pacific Islander (I: 29%, C: 29%), Maori (I: 13%, C: 14%), New Zealand European or other (I: 58%, C: 57%) | I: 31.6 ± 4.6  C: 31.1 ± 5.2 | 20 weeks | I: 37  C: 37 | OB/OW | Mobile application  At home | PA (moderate stationary cycling) | 67 home-based exercises (3 to 5 times/week, 15 to 30 min/session) | From 20 to 35 gestation weeks | Routine physical  activity | Birth outcomes  GWG |
| Smith  (2016) | NR | I: 29.4 ± 4.9  C: 29.7 ± 4.1 | 10 - 14 weeks | I: 21  C: 22 | OB | In community | PA (moderate)  B (PA and D) | PA (150 min/week) | Up to 36 gestation weeks | E (one-time advice session) | GWG  PAL |
| Thomas  (2022) | Asian (I: 15%, C: 14%),  White (I: 55%, C: 63%),  Hispanic (I: 9%, C: 11%),  African American (I: 3%, C: 3%),  Multiracial or other (I: 18%, C: 9%) | I: 34.8 ± 4.2  C: 33.2 ± 3.7 | I: 11.0 ±1.8  C: 10.7 ±1.3 | I: 35  C: 33 | OB/OW | Mobile application  At clinic | B (PA and D) | 2 in-person and 11 telephone sessions (NR) | 3 months | Routine prenatal care | GWG |
| Thornton (2009) | African American (I: 39.7%, C: 42.2%), Caucasian (I: 21.6%, C: 23.3%), Latina (I: 25%, C: 21.6%),  Indian (I: 13.7%, C: 12.9%) | I: 26.8 (median)  C: 27.3 (median) | During prenatal care | I: 116  C: 116 | OB | At clinic | D (18 - 24 kcal/kg; 40% carbohydrates, 30% protein, and 30% fat) | Individual sessions at  prenatal care (NR) | During prenatal care | Routine prenatal care | GWG  Gestational diabetes  Preeclampsia  Gestational hypertension  Ketonuria |
| Vesco (2014) | White (I: 88%, C: 85%) | I: 32.4 ± 5.1  C: 31.2 ± 4.6 | I: 14.6 ± 2.8  C: 15.1 ± 2.5 | I: 57  C: 55 | OB | At research facility | PA (moderate),  D (30 kcal/kg/day reduced by 30%)  B (PA and D) | Two individual counseling sessions (NR)  Weekly group sessions (90 min) | From 10 gestation weeks to term | E (received onetime dietary advice) | GWG  Newborn outcome |
| Vinter (2011) | Caucasian (I: 100%, C: 100%) | I: 29  C: 29  (Median) | 10 - 14 weeks | I: 150  C: 154 | OB | At clinic | PA (Moderate),  B (PA and D) | 4 individual counseling sessions  Weekly training classes (1 hour/session) | 16 weeks | E (PA and D) | GWG  Preeclampsia, PIH  GDM, Cesarean section |
| Willcox  (2017) | Australia (I: 74%, C: 80%),  Other (I: 26%, C: 20%) | I: 32.0 ± 5.1  C: 33.0 ± 3.4 | I: 15.8 ± 1.7 C: 15.2 ± 2.3 | I:46  C:45 | OB/OW | At hospital | PA (Moderate)  D (Australian Dietary  Guidelines)  E (Advice  regarding diet and physical activity) | weekly or fortnightly (30 min/session) | Up to 36 gestation weeks | E (Advice  regarding diet and physical activity) | GWG |
| Wolff (2008) | Caucasian (I: 100%, C: 100%) | I: 28.0 ± 4.0  C: 30.0 ± 5.0 | I: 15.0 ± 2.0  C: 16.0 ± 3.0 | I: 23  C: 27 | OB | At clinic | D (30% fat,  20% protein, and 50% carbohydrate)  B (D) | 10 counseling sessions (1 hour/session) | Up to 36 gestation weeks | Routine prenatal care | GWG  Glucose metabolism  Birth outcomes |

MGS: maternal glucose screen; GDM: gestational diabetes mellitus; RMR: resting metabolic rating, PAL: physical activity level; OGTT: oral glucose tolerance test; VO_2_: the values of oxygen uptake at the anaerobic threshold; PIH: pregnancy-induced hypertension; NR: no report; OW: overweight; OB: obesity; I: intervention group; C: control group; T: total; PA: physical activity; D: dietary; B: behavior.

**Supplementary Figure. 1** Risk of Bias graph


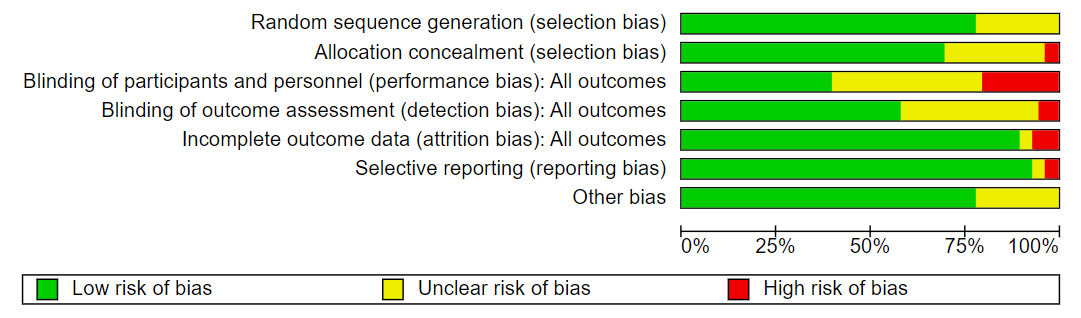


**Supplementary Figure. 2 Risk of bias summary**


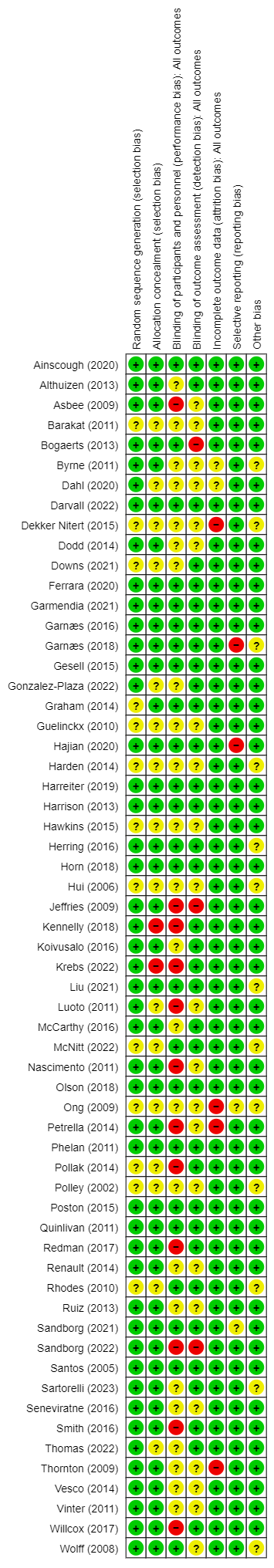


**Supplementary Table 4** The confidence rating for mean overall change in GWG by CINeMA.

| **Comparison** | **No. of studies** | **Within-study bias** | **Reporting bias** | **Indirectness** | **Imprecision** | **Heterogeneity** | **Incoherence** | **Confidence rating** |
| --- | --- | --- | --- | --- | --- | --- | --- | --- |
| I-B vs. IB + R-B(m) | 1 | Some concerns | Low risk | No concerns | No concerns | Some  concerns | No concerns | Low |
| I-B vs. I-DB | 1 | Some concerns | Low risk | No concerns | No concerns | No concerns | No concerns | Moderate |
| I-B vs. I-E | 1 | Some concerns | Low risk | No concerns | No concerns | No concerns | No concerns | Moderate |
| I-B vs. I-PAB | 1 | Some concerns | Low risk | No concerns | No concerns | No concerns | No concerns | Moderate |
| I-B vs. I-PADB | 2 | Some concerns | Low risk | No concerns | No concerns | Some  concerns | No concerns | Low |
| I-B vs. Placebo | 16 | Some concerns | Some concerns | No concerns | No concerns | No concerns | No concerns | Low |
| I-B vs. RPADB(m) | 1 | Some concerns | Some concerns | No concerns | No concerns | No concerns | No concerns | Low |
| I-B + R-B(e) vs. Placebo | 2 | Some concerns | Low risk | No concerns | No concerns | No concerns | No concerns | Moderate |
| I-B + R-B(m) vs. Placebo | 1 | Major concerns | Low risk | No concerns | Some concerns | No concerns | No concerns | Very low |
| I-B + R-DB(e) vs. IE | 1 | No concerns | Low risk | No concerns | No concerns | No concerns | No concerns | High |
| I-D vs. Placebo | 1 | Major concerns | Low risk | Major concerns | No concerns | No concerns | No concerns | Very low |
| I-DB vs. Placebo | 4 | Some concerns | Low risk | No concerns | No concerns | No concerns | No concerns | Moderate |
| I-DB + R-B (e) vs. Placebo | 1 | Some concerns | Low risk | No concerns | No concerns | Some  concerns | No concerns | Low |
| I-E vs. I-E + R-PA(m) | 1 | Some concerns | Low risk | No concerns | Some concerns | No concerns | No concerns | Low |
| I-E vs. I-PA | 1 | Some concerns | Low risk | No concerns | No concerns | No concerns | No concerns | Moderate |
| I-E vs. I-PAB | 1 | Some concerns | Low risk | No concerns | No concerns | Some  concerns | No concerns | Low |
| I-E vs. I-PAD | 2 | Some concerns | Low risk | Some concerns | No concerns | Some  concerns | No concerns | Very low |
| I-E vs. I-PADB | 1 | Some concerns | Low risk | No concerns | No concerns | Some  concerns | No concerns | Low |
| I-E vs. I-PADB + R-PAB(e) | 1 | Some concerns | Low risk | No concerns | No concerns | Some  concerns | No concerns | Low |
| I-E vs. I-PAE | 1 | Major concerns | Low risk | Major concerns | No concerns | Major  concerns | No concerns | Very low |
| I-E vs. Placebo | 5 | Some concerns | Low risk | No concerns | No concerns | No concerns | No concerns | Moderate |
| I-E vs. R-PAB(e) | 1 | Major concerns | Low risk | No concerns | Some concerns | No concerns | No concerns | Very low |
| I-PA vs. I-PAD | 1 | Some concerns | Low risk | No concerns | No concerns | Some  concerns | No concerns | Low |
| I-PA vs. Placebo | 8 | Some concerns | Major concerns | No concerns | No concerns | No concerns | No concerns | Very low |
| I-PAB vs. Placebo | 4 | Some concerns | Low risk | No concerns | No concerns | Some  concerns | No concerns | Low |
| I-PAD vs. Placebo | 1 | Some concerns | Low risk | No concerns | No concerns | Some  concerns | No concerns | Low |
| I-PADB vs. Placebo | 1 | Some concerns | Low risk | No concerns | No concerns | Some  concerns | Major concerns | Very low |
| I-PADB + R-B(e) vs. Placebo | 1 | Some concerns | Low risk | No concerns | No concerns | Some concerns | No concerns | Low |
| Placebo vs. R-B(e) | 6 | Some concerns | Low risk | No concerns | No concerns | No concerns | No concerns | Moderate |
| Placebo vs. R-B(m) | 1 | Some concerns | Low risk | No concerns | No concerns | No concerns | No concerns | Moderate |
| Placebo vs. R-B(m+e) | 1 | Some concerns | Low risk | No concerns | No concerns | Some  concerns | No concerns | Low |
| Placebo vs. R-PA(m+e) | 1 | Some concerns | Low risk | No concerns | No concerns | Some  concerns | No concerns | Low |
| Placebo vs. R-PADB(e) | 1 | Some concerns | Low risk | No concerns | Some concerns | No concerns | No concerns | Low |
| R-B(e) vs. R-E(e) | 1 | Major concerns | Low risk | No concerns | No concerns | Major  concerns | No concerns | Very low |
| R-B(e) vs. R-PADB(e) | 1 | Some concerns | Low risk | No concerns | No concerns | Some  concerns | No concerns | Low |
| R-B(m) vs. R-PADB(m) | 1 | Major concerns | Low risk | No concerns | No concerns | Some  concerns | No concerns | Very low |
| R-E(e) vs. R-PADE(e) | 1 | Major concerns | Low risk | No concerns | Some concerns | No concerns | No concerns | Very low |

R, remote; I, in-person; e, electronic heath; m, mobile health; PA, physical activity; D, dietary; E, education; B, behavior.

**Supplementary Table 5** Results of local inconsistency analysis for all direct and indirect comparisons.

| **Comparison** | **Direct** | | **Indirect** | | **Difference** | |  |  |
| --- | --- | --- | --- | --- | --- | --- | --- | --- |
|  | **Coef.** | **Std. Err.** | **Coef.** | **Std. Err.** | **Coef.** | **Std. Err.** | **P>z** | **tau** |
| I-B vs. I-B + RB(m) | -0.9942449 | 0.665257 | 0.1324175 | 0.4833149 | -1.126662 | 0.8222896 | 0.171 | 0.460622 |
| I-B vs. I-DB | -0.1591406 | 0.521936 | 0.2065535 | 0.307021 | -0.3656941 | 0.6059312 | 0.546 | 0.470618 |
| I-B vs. I-E | -0.0815064 | 0.5001416 | 0.3407803 | 0.2247791 | -0.4222866 | 0.5480988 | 0.441 | 0.4687697 |
| I-B vs. I-PAB | 0.3975449 | 0.5934294 | -0.1578448 | 0.2697547 | 0.5553896 | 0.6518635 | 0.394 | 0.4691403 |
| I-B vs. I-PADB | -0.8655292 | 0.4682661 | 0.1784074 | 0.3957824 | -1.043937 | 0.6131077 | 0.089 | 0.4571633 |
| I-B vs. Placebo | 0.4086587 | 0.1268311 | -0.1105702 | 0.3165694 | 0.5192289 | 0.3409068 | 0.128 | 0.4575085 |
| I-B vs. RPADB(m) | -0.0623084 | 0.4921843 | -0.0888317 | 0.6995153 | 0.0265233 | 0.8553169 | 0.975 | 0.4754374 |
| I-B + RB(e) vs. Placebo | 0.1414594 | 0.3369665 | 0.6847633 | 44.72312 | -0.5433039 | 44.72438 | 0.99 | 0.4655151 |
| I-B + RB(m) vs. Placebo | 0.2288608 | 0.4682959 | 1.355487 | 0.6758914 | -1.126626 | 0.8222714 | 0.171 | 0.4606223 |
| I-B + RDB(e) vs. IE | 0.0552282 | 0.4892017 | 0.5412504 | 63.25258 | -0.4860222 | 63.25446 | 0.994 | 0.4655148 |
| I-D vs. Placebo | 1.272992 | 0.4873507 | 0.6761821 | 63.24811 | 0.5968102 | 63.24999 | 0.992 | 0.4655146 |
| I-DB vs. Placebo | 0.1747855 | 0.2524346 | 0.9310754 | 0.9361352 | -0.7562899 | 0.9692235 | 0.435 | 0.468985 |
| I-DB + RB (e) vs. Placebo | 0.1117496 | 0.5699531 | 0.6585525 | 63.25069 | -0.546803 | 63.25353 | 0.993 | 0.465515 |
| I-E vs. I-E + R-PA (m) | -0.4180843 | 0.5030368 | -0.514395 | 63.25303 | 0.0963107 | 63.25506 | 0.999 | 0.4655148 |
| I-E vs. I-PA | -0.1172284 | 0.490106 | -0.0674408 | 0.2726455 | -0.0497876 | 0.5608382 | 0.929 | 0.4749497 |
| I-E vs. I-PAB | -1.091372 | 0.4501186 | -0.0091763 | 0.2930453 | -1.082196 | 0.5371055 | 0.044 | 0.4329624 |
| I-E vs. I-PAD | -0.3545965 | 0.427354 | -0.2993356 | 0.4681627 | -0.0552609 | 0.6338365 | 0.931 | 0.4737124 |
| I-E vs. I-PADB | -0.7646592 | 0.5085693 | -0.3750794 | 0.409608 | -0.3895798 | 0.6530095 | 0.551 | 0.4692454 |
| I-E vs. I-PADB + RPAB(e) | 0.1103958 | 0.5533002 | -0.5476797 | 63.2588 | 0.6580755 | 63.26132 | 0.992 | 0.4655148 |
| I-E vs. I-PAE | -0.0790036 | 0.5761413 | -0.5335386 | 63.24628 | 0.454535 | 63.24906 | 0.994 | 0.4655149 |
| I-E vs. Placebo | 0.2953239 | 0.2248071 | -0.2602153 | 0.2679353 | 0.5555393 | 0.3495792 | 0.112 | 0.4466395 |
| I-E vs. R-PAB(e) | 0.4391341 | 0.5588362 | -0.5505458 | 63.25956 | 0.9896799 | 63.26206 | 0.988 | 0.4655147 |
| I-PA vs. I-PAD | -0.1546531 | 0.4896222 | -0.3373958 | 0.4676835 | 0.1827427 | 0.6771362 | 0.787 | 0.4732932 |
| I-PA vs. Placebo | 0.1387518 | 0.2077539 | 0.2073009 | 0.5073783 | -0.0685491 | 0.5480792 | 0.9 | 0.4747023 |
| I-PAB vs. Placebo | 0.253141 | 0.2701667 | 0.706015 | 0.3895318 | -0.452874 | 0.4759407 | 0.341 | 0.4580536 |
| I-PAD vs. Placebo | 0.4445845 | 0.4899505 | 0.3644415 | 0.4237994 | 0.0801429 | 0.647766 | 0.902 | 0.4739607 |
| I-PADB vs. Placebo | -0.5083611 | 0.5238875 | 1.066483 | 0.3473458 | -1.574844 | 0.6285759 | 0.012 | 0.4347951 |
| I-PADB + RB(e) vs. Placebo | 0.1696879 | 0.5268777 | 0.6813569 | 63.25325 | -0.511669 | 63.25542 | 0.994 | 0.465514 |
| Placebo vs. R-B(e) | -0.1225373 | 0.2024972 | -0.214219 | 0.7333389 | 0.0916817 | 0.7607831 | 0.904 | 0.474215 |
| Placebo vs. R-B(m) | 0.0118823 | 0.4829744 | 0.0381696 | 0.7058824 | -0.0262873 | 0.8552977 | 0.975 | 0.4754372 |
| Placebo vs. R-B(m+e) | 0.1498561 | 0.5251247 | -0.6717908 | 63.25391 | 0.8216469 | 63.25606 | 0.99 | 0.4655147 |
| Placebo vs. R-PA(m+e) | -0.213703 | 0.5206608 | -0.676196 | 63.25415 | 0.462493 | 63.25629 | 0.994 | 0.4655147 |
| Placebo vs. R-PADB(e) | -0.546211 | 0.5471072 | -0.4550174 | 0.5297728 | -0.0911936 | 0.7615675 | 0.905 | 0.474232 |
| R-B(e) vs. R-E(e) | 0.0395322 | 0.5861264 | -0.0757532 | 28.28767 | 0.1152854 | 28.29346 | 0.997 | 0.4655181 |
| R-B(e) vs. R-PADB(e) | -0.3324098 | 0.489595 | -0.4236398 | 0.5833438 | 0.0912299 | 0.7615729 | 0.905 | 0.4742325 |
| R-B(m) vs. R-PADB(m) | -0.4391733 | 0.4910873 | -0.4126989 | 0.7003871 | -0.0264744 | 0.8553998 | 0.975 | 0.4754393 |
| R-E(e) vs. R-PADE(e) | -0.4366303 | 0.5116374 | -0.5020502 | 63.26593 | 0.0654199 | 63.26799 | 0.999 | 0.4655172 |

R, remote; I, in-person; e, electronic heath; m, mobile health; PA, physical activity; D, dietary; E, education; B, behavior.

**The list of included studies ([1-60]).**

1. Ainscough, K.M.; O'Brien, E.C.; Lindsay, K.L.; Kennelly, M.A.; O'Sullivan, E.J.; O'Brien, O.A.; McCarthy, M.; De Vito, G.; McAuliffe, F.M. Nutrition, Behavior Change and Physical Activity Outcomes From the PEARS RCT—An mHealth-Supported, Lifestyle Intervention Among Pregnant Women With Overweight and Obesity. *Frontiers in Endocrinology* **2020**, *10*, doi:10.3389/fendo.2019.00938.

2. Althuizen, E.; van der Wijden, C.L.; van Mechelen, W.; Seidell, J.C.; van Poppel, M.N. The effect of a counselling intervention on weight changes during and after pregnancy: a randomised trial. *Bjog* **2013**, *120*, 92-99, doi:10.1111/1471-0528.12014.

3. Asbee, S.M.; Jenkins, T.R.; Butler, J.R.; White, J.; Elliot, M.; Rutledge, A. Preventing excessive weight gain during pregnancy through dietary and lifestyle counseling: a randomized controlled trial. *Obstet Gynecol* **2009**, *113*, 305-312, doi:10.1097/AOG.0b013e318195baef.

4. Ruben, B.; Yaiza, C.; Javier, C.; Maria, L.; Rocio, M. Exercise during pregnancy improves maternal glucose screen at 24–28 weeks: a randomised controlled trial. *British Journal of Sports Medicine* **2011**, bjsports-2011-090009, doi:10.1136/bjsports-2011-090009.

5. Bogaerts, A.F.L.; Devlieger, R.; Nuyts, E.; Witters, I.; Gyselaers, W.; Van den Bergh, B.R.H. Effects of lifestyle intervention in obese pregnant women on gestational weight gain and mental health: a randomized controlled trial. *International Journal of Obesity* **2013**, *37*, 814-821, doi:10.1038/ijo.2012.162.

6. Byrne, N.M.; Groves, A.M.; McIntyre, H.D.; Callaway, L.K. Changes in resting and walking energy expenditure and walking speed during pregnancy in obese women. *Am J Clin Nutr* **2011**, *94*, 819-830, doi:10.3945/ajcn.110.009399.

7. Dahl, A.A. Healthy Motivations for Moms-To-Be (Healthy MoM2B) Study: A mobile health intervention targeting gestational weight gain among US women. University of South Carolina, 2018.

8. Darvall, J.N.; Wang, A.; Nazeem, M.N.; Harrison, C.L.; Clarke, L.; Mendoza, C.; Parker, A.; Harrap, B.; Teale, G.; Story, D.; et al. A Pedometer-Guided Physical Activity Intervention for Obese Pregnant Women (the Fit MUM Study): Randomized Feasibility Study. *JMIR Mhealth Uhealth* **2020**, *8*, e15112, doi:10.2196/15112.

9. Dekker Nitert, M.; Barrett, H.L.; Denny, K.J.; McIntyre, H.D.; Callaway, L.K.; the, B.g. Exercise in pregnancy does not alter gestational weight gain, MCP-1 or leptin in obese women. *Australian and New Zealand Journal of Obstetrics and Gynaecology* **2015**, *55*, 27-33, doi:https://doi.org/10.1111/ajo.12300.

10. Dodd, J.M.; Cramp, C.; Sui, Z.; Yelland, L.N.; Deussen, A.R.; Grivell, R.M.; Moran, L.J.; Crowther, C.A.; Turnbull, D.; McPhee, A.J.; et al. The effects of antenatal dietary and lifestyle advice for women who are overweight or obese on maternal diet and physical activity: the LIMIT randomised trial. *BMC Medicine* **2014**, *12*, 161, doi:10.1186/s12916-014-0161-y.

11. Downs, D.S.; Savage, J.S.; Rivera, D.E.; Pauley, A.M.; Leonard, K.S.; Hohman, E.E.; Guo, P.; McNitt, K.M.; Stetter, C.; Kunselman, A. Adaptive, behavioral intervention impact on weight gain, physical activity, energy intake, and motivational determinants: results of a feasibility trial in pregnant women with overweight/obesity. *Journal of Behavioral Medicine* **2021**, *44*, 605-621, doi:10.1007/s10865-021-00227-9.

12. Ferrara, A.; Hedderson, M.M.; Brown, S.D.; Ehrlich, S.F.; Tsai, A.-L.; Feng, J.; Galarce, M.; Marcovina, S.; Catalano, P.; Quesenberry, C.P. A telehealth lifestyle intervention to reduce excess gestational weight gain in pregnant women with overweight or obesity (GLOW): a randomised, parallel-group, controlled trial. *The Lancet Diabetes & Endocrinology* **2020**, *8*, 490-500, doi:10.1016/S2213-8587(20)30107-8.

13. Garmendia, M.L.; Casanello, P.; Flores, M.; Kusanovic, J.P.; Uauy, R. The effects of a combined intervention (docosahexaenoic acid supplementation and home-based dietary counseling) on metabolic control in obese and overweight pregnant women: the MIGHT study. *American Journal of Obstetrics and Gynecology* **2021**, *224*, 526.e521-526.e525, doi:https://doi.org/10.1016/j.ajog.2020.10.048.

14. Garnæs, K.K.; Mørkved, S.; Salvesen, Ø.; Moholdt, T. Exercise Training and Weight Gain in Obese Pregnant Women: A Randomized Controlled Trial (ETIP Trial). *PLOS Medicine* **2016**, *13*, e1002079, doi:10.1371/journal.pmed.1002079.

15. Garnæs, K.K.; Mørkved, S.; Salvesen, K.Å.; Salvesen, Ø.; Moholdt, T. Exercise training during pregnancy reduces circulating insulin levels in overweight/obese women postpartum: secondary analysis of a randomised controlled trial (the ETIP trial). *BMC Pregnancy and Childbirth* **2018**, *18*, 18, doi:10.1186/s12884-017-1653-5.

16. Gesell, S.B.; Katula, J.A.; Strickland, C.; Vitolins, M.Z. Feasibility and Initial Efficacy Evaluation of a Community-Based Cognitive-Behavioral Lifestyle Intervention to Prevent Excessive Weight Gain During Pregnancy in Latina Women. *Maternal and Child Health Journal* **2015**, *19*, 1842-1852, doi:10.1007/s10995-015-1698-x.

17. Gonzalez-Plaza, E.; Bellart, J.; Arranz, Á.; Luján-Barroso, L.; Crespo Mirasol, E.; Seguranyes, G. Effectiveness of a Step Counter Smartband and Midwife Counseling Intervention on Gestational Weight Gain and Physical Activity in Pregnant Women With Obesity (Pas and Pes Study): Randomized Controlled Trial. *JMIR Mhealth Uhealth* **2022**, *10*, e28886, doi:10.2196/28886.

18. Graham, M.L.; Uesugi, K.H.; Niederdeppe, J.; Gay, G.K.; Olson, C.M. The Theory, Development, and Implementation of an e-Intervention to Prevent Excessive Gestational Weight Gain: e-Moms Roc. *Telemedicine and e-Health* **2014**, *20*, 1135-1142, doi:10.1089/tmj.2013.0354.

19. Guelinckx, I.; Devlieger, R.; Mullie, P.; Vansant, G. Effect of lifestyle intervention on dietary habits, physical activity, and gestational weight gain in obese pregnant women: a randomized controlled trial. *Am J Clin Nutr* **2010**, *91*, 373-380, doi:10.3945/ajcn.2009.28166.

20. Hajian, S.; Aslani, A.; Sarbakhsh, P.; Fathnezhad-Kazemi, A. The effectiveness of healthy lifestyle interventions on weight gain in overweight pregnant women: A cluster-randomized controlled trial. *Nurs Open* **2020**, *7*, 1876-1886, doi:10.1002/nop2.577.

21. Harden, S.M.; Beauchamp, M.R.; Pitts, B.H.; Nault, E.M.; Davy, B.M.; You, W.; Weiss, P.; Estabrooks, P.A. Group-based lifestyle sessions for gestational weight gain management: a mixed method approach. *Am J Health Behav* **2014**, *38*, 560-569, doi:10.5993/ajhb.38.4.9.

22. Harreiter, J.; Simmons, D.; Desoye, G.; Corcoy, R.; Adelantado, J.M.; Devlieger, R.; Galjaard, S.; Damm, P.; Mathiesen, E.R.; Jensen, D.M.; et al. Nutritional Lifestyle Intervention in Obese Pregnant Women, Including Lower Carbohydrate Intake, Is Associated With Increased Maternal Free Fatty Acids, 3-β-Hydroxybutyrate, and Fasting Glucose Concentrations: A Secondary Factorial Analysis of the European Multicenter, Randomized Controlled DALI Lifestyle Intervention Trial. *Diabetes Care* **2019**, *42*, 1380-1389, doi:10.2337/dc19-0418.

23. Harrison, C.L.; Lombard, C.B.; Strauss, B.J.; Teede, H.J. Optimizing healthy gestational weight gain in women at high risk of gestational diabetes: a randomized controlled trial. *Obesity (Silver Spring)* **2013**, *21*, 904-909, doi:10.1002/oby.20163.

24. Hawkins, M.; Hosker, M.; Marcus, B.H.; Rosal, M.C.; Braun, B.; Stanek, E.J., 3rd; Markenson, G.; Chasan-Taber, L. A pregnancy lifestyle intervention to prevent gestational diabetes risk factors in overweight Hispanic women: a feasibility randomized controlled trial. *Diabet Med* **2015**, *32*, 108-115, doi:10.1111/dme.12601.

25. Herring, S.J.; Cruice, J.F.; Bennett, G.G.; Rose, M.Z.; Davey, A.; Foster, G.D. Preventing excessive gestational weight gain among African American women: A randomized clinical trial. *Obesity (Silver Spring)* **2016**, *24*, 30-36, doi:10.1002/oby.21240.

26. Van Horn, L.; Peaceman, A.; Kwasny, M.; Vincent, E.; Fought, A.; Josefson, J.; Spring, B.; Neff, L.M.; Gernhofer, N. Dietary Approaches to Stop Hypertension Diet and Activity to Limit Gestational Weight: Maternal Offspring Metabolics Family Intervention Trial, a Technology Enhanced Randomized Trial. *Am J Prev Med* **2018**, *55*, 603-614, doi:10.1016/j.amepre.2018.06.015.

27. Hui, A.L.; Ludwig, S.; Gardiner, P.; Sevenhuysen, G.; Murray, R.; Morris, M.; Shen, G.X. Community-based Exercise and Dietary Intervention During Pregnancy:A Pilot Study. *Canadian Journal of Diabetes* **2006**, *30*, 1-7, doi:https://doi.org/10.1016/S1499-2671(06)02010-7.

28. Jeffries, K.; Shub, A.; Walker, S.P.; Hiscock, R.; Permezel, M. Reducing excessive weight gain in pregnancy: a randomised controlled trial. *Med J Aust* **2009**, *191*, 429-433, doi:10.5694/j.1326-5377.2009.tb02877.x.

29. Kennelly, M.A.; Ainscough, K.; Lindsay, K.L.; O'Sullivan, E.; Gibney, E.R.; McCarthy, M.; Segurado, R.; DeVito, G.; Maguire, O.; Smith, T.; et al. Pregnancy Exercise and Nutrition With Smartphone Application Support: A Randomized Controlled Trial. *Obstet Gynecol* **2018**, *131*, 818-826, doi:10.1097/aog.0000000000002582.

30. Koivusalo, S.B.; Rönö, K.; Klemetti, M.M.; Roine, R.P.; Lindström, J.; Erkkola, M.; Kaaja, R.J.; Pöyhönen-Alho, M.; Tiitinen, A.; Huvinen, E.; et al. Gestational Diabetes Mellitus Can Be Prevented by Lifestyle Intervention: The Finnish Gestational Diabetes Prevention Study (RADIEL): A Randomized Controlled Trial. *Diabetes Care* **2016**, *39*, 24-30, doi:10.2337/dc15-0511.

31. Krebs, F.; Lorenz, L.; Nawabi, F.; Alayli, A.; Stock, S. Effectiveness of a Brief Lifestyle Intervention in the Prenatal Care Setting to Prevent Excessive Gestational Weight Gain and Improve Maternal and Infant Health Outcomes. *Int J Environ Res Public Health* **2022**, *19*, doi:10.3390/ijerph19105863.

32. Liu, J.; Wilcox, S.; Wingard, E.; Turner-McGrievy, G.; Hutto, B.; Burgis, J. A Behavioral Lifestyle Intervention to Limit Gestational Weight Gain in Pregnant Women with Overweight and Obesity. *Obesity (Silver Spring)* **2021**, *29*, 672-680, doi:10.1002/oby.23119.

33. Luoto, R.; Kinnunen, T.I.; Aittasalo, M.; Kolu, P.; Raitanen, J.; Ojala, K.; Mansikkamäki, K.; Lamberg, S.; Vasankari, T.; Komulainen, T.; et al. Primary prevention of gestational diabetes mellitus and large-for-gestational-age newborns by lifestyle counseling: a cluster-randomized controlled trial. *PLoS Med* **2011**, *8*, e1001036, doi:10.1371/journal.pmed.1001036.

34. McCarthy, E.A.; Walker, S.P.; Ugoni, A.; Lappas, M.; Leong, O.; Shub, A. Self-weighing and simple dietary advice for overweight and obese pregnant women to reduce obstetric complications without impact on quality of life: a randomised controlled trial. *Bjog* **2016**, *123*, 965-973, doi:10.1111/1471-0528.13919.

35. McNitt, K.M.; Hohman, E.E.; Rivera, D.E.; Guo, P.; Pauley, A.M.; Gernand, A.D.; Symons Downs, D.; Savage, J.S. Underreporting of Energy Intake Increases over Pregnancy: An Intensive Longitudinal Study of Women with Overweight and Obesity. *Nutrients* **2022**, *14*, doi:10.3390/nu14112326.

36. Nascimento, S.L.; Surita, F.G.; Parpinelli, M.; Siani, S.; Pinto e Silva, J.L. The effect of an antenatal physical exercise programme on maternal/perinatal outcomes and quality of life in overweight and obese pregnant women: a randomised clinical trial. *Bjog* **2011**, *118*, 1455-1463, doi:10.1111/j.1471-0528.2011.03084.x.

37. Olson, C.M.; Groth, S.W.; Graham, M.L.; Reschke, J.E.; Strawderman, M.S.; Fernandez, I.D. The effectiveness of an online intervention in preventing excessive gestational weight gain: the e-moms roc randomized controlled trial. *BMC Pregnancy and Childbirth* **2018**, *18*, 148, doi:10.1186/s12884-018-1767-4.

38. Ong, M.J.; Guelfi, K.J.; Hunter, T.; Wallman, K.E.; Fournier, P.A.; Newnham, J.P. Supervised home-based exercise may attenuate the decline of glucose tolerance in obese pregnant women. *Diabetes Metab* **2009**, *35*, 418-421, doi:10.1016/j.diabet.2009.04.008.

39. Petrella, E.; Malavolti, M.; Bertarini, V.; Pignatti, L.; Neri, I.; Battistini, N.C.; Facchinetti, F. Gestational weight gain in overweight and obese women enrolled in a healthy lifestyle and eating habits program. *J Matern Fetal Neonatal Med* **2014**, *27*, 1348-1352, doi:10.3109/14767058.2013.858318.

40. Phelan, S.; Phipps, M.G.; Abrams, B.; Darroch, F.; Schaffner, A.; Wing, R.R. Randomized trial of a behavioral intervention to prevent excessive gestational weight gain: the Fit for Delivery Study. *Am J Clin Nutr* **2011**, *93*, 772-779, doi:10.3945/ajcn.110.005306.

41. Pollak, K.I.; Alexander, S.C.; Bennett, G.; Lyna, P.; Coffman, C.J.; Bilheimer, A.; Farrell, D.; Bodner, M.E.; Swamy, G.K.; Østbye, T. Weight-related SMS texts promoting appropriate pregnancy weight gain: a pilot study. *Patient Educ Couns* **2014**, *97*, 256-260, doi:10.1016/j.pec.2014.07.030.

42. Polley, B.A.; Wing, R.R.; Sims, C.J. Randomized controlled trial to prevent excessive weight gain in pregnant women. *Int J Obes Relat Metab Disord* **2002**, *26*, 1494-1502, doi:10.1038/sj.ijo.0802130.

43. Poston, L.; Bell, R.; Croker, H.; Flynn, A.C.; Godfrey, K.M.; Goff, L.; Hayes, L.; Khazaezadeh, N.; Nelson, S.M.; Oteng-Ntim, E.; et al. Effect of a behavioural intervention in obese pregnant women (the UPBEAT study): a multicentre, randomised controlled trial. *Lancet Diabetes Endocrinol* **2015**, *3*, 767-777, doi:10.1016/s2213-8587(15)00227-2.

44. Quinlivan, J.A.; Lam, L.T.; Fisher, J. A randomised trial of a four-step multidisciplinary approach to the antenatal care of obese pregnant women. *Aust N Z J Obstet Gynaecol* **2011**, *51*, 141-146, doi:10.1111/j.1479-828X.2010.01268.x.

45. Redman, L.M.; Gilmore, L.A.; Breaux, J.; Thomas, D.M.; Elkind-Hirsch, K.; Stewart, T.; Hsia, D.S.; Burton, J.; Apolzan, J.W.; Cain, L.E.; et al. Effectiveness of SmartMoms, a Novel eHealth Intervention for Management of Gestational Weight Gain: Randomized Controlled Pilot Trial. *JMIR Mhealth Uhealth* **2017**, *5*, e133, doi:10.2196/mhealth.8228.

46. Renault, K.M.; Nørgaard, K.; Nilas, L.; Carlsen, E.M.; Cortes, D.; Pryds, O.; Secher, N.J. The Treatment of Obese Pregnant Women (TOP) study: a randomized controlled trial of the effect of physical activity intervention assessed by pedometer with or without dietary intervention in obese pregnant women. *Am J Obstet Gynecol* **2014**, *210*, 134.e131-139, doi:10.1016/j.ajog.2013.09.029.

47. Rhodes, E.T.; Pawlak, D.B.; Takoudes, T.C.; Ebbeling, C.B.; Feldman, H.A.; Lovesky, M.M.; Cooke, E.A.; Leidig, M.M.; Ludwig, D.S. Effects of a low-glycemic load diet in overweight and obese pregnant women: a pilot randomized controlled trial. *Am J Clin Nutr* **2010**, *92*, 1306-1315, doi:10.3945/ajcn.2010.30130.

48. Ruiz, J.R.; Perales, M.; Pelaez, M.; Lopez, C.; Lucia, A.; Barakat, R. Supervised exercise-based intervention to prevent excessive gestational weight gain: a randomized controlled trial. *Mayo Clin Proc* **2013**, *88*, 1388-1397, doi:10.1016/j.mayocp.2013.07.020.

49. Sandborg, J.; Söderström, E.; Henriksson, P.; Bendtsen, M.; Henström, M.; Leppänen, M.H.; Maddison, R.; Migueles, J.H.; Blomberg, M.; Löf, M. Effectiveness of a Smartphone App to Promote Healthy Weight Gain, Diet, and Physical Activity During Pregnancy (HealthyMoms): Randomized Controlled Trial. *JMIR Mhealth Uhealth* **2021**, *9*, e26091, doi:10.2196/26091.

50. Sandborg, J.; Henriksson, P.; Söderström, E.; Migueles, J.H.; Bendtsen, M.; Blomberg, M.; Löf, M. The effects of a lifestyle intervention (the HealthyMoms app) during pregnancy on infant body composition: Secondary outcome analysis from a randomized controlled trial. *Pediatr Obes* **2022**, *17*, e12894, doi:10.1111/ijpo.12894.

51. Santos, I.A.; Stein, R.; Fuchs, S.C.; Duncan, B.B.; Ribeiro, J.P.; Kroeff, L.R.; Carballo, M.T.; Schmidt, M.I. Aerobic exercise and submaximal functional capacity in overweight pregnant women: a randomized trial. *Obstet Gynecol* **2005**, *106*, 243-249, doi:10.1097/01.Aog.0000171113.36624.86.

52. Sartorelli, D.S.; Crivellenti, L.C.; Baroni, N.F.; de Andrade Miranda, D.E.G.; da Silva Santos, I.; Carvalho, M.R.; de Lima, M.C.; Carreira, N.P.; Chaves, A.V.L.; Manochio-Pina, M.G.; et al. Effectiveness of a minimally processed food-based nutritional counselling intervention on weight gain in overweight pregnant women: a randomized controlled trial. *Eur J Nutr* **2023**, *62*, 443-454, doi:10.1007/s00394-022-02995-9.

53. Seneviratne, S.N.; Jiang, Y.; Derraik, J.; McCowan, L.; Parry, G.K.; Biggs, J.B.; Craigie, S.; Gusso, S.; Peres, G.; Rodrigues, R.O.; et al. Effects of antenatal exercise in overweight and obese pregnant women on maternal and perinatal outcomes: a randomised controlled trial. *Bjog* **2016**, *123*, 588-597, doi:10.1111/1471-0528.13738.

54. Smith, K.; Lanningham-Foster, L.; Welch, A.; Campbell, C. Web-Based Behavioral Intervention Increases Maternal Exercise but Does Not Prevent Excessive Gestational Weight Gain in Previously Sedentary Women. *J Phys Act Health* **2016**, *13*, 587-593, doi:10.1123/jpah.2015-0219.

55. Thomas, T.; Xu, F.; Sridhar, S.; Sedgwick, T.; Nkemere, L.; Badon, S.E.; Quesenberry, C.; Ferrara, A.; Mandel, S.; Brown, S.D.; et al. A Web-Based mHealth Intervention With Telephone Support to Increase Physical Activity Among Pregnant Patients With Overweight or Obesity: Feasibility Randomized Controlled Trial. *JMIR Form Res* **2022**, *6*, e33929, doi:10.2196/33929.

56. Thornton, Y.S.; Smarkola, C.; Kopacz, S.M.; Ishoof, S.B. Perinatal outcomes in nutritionally monitored obese pregnant women: a randomized clinical trial. *J Natl Med Assoc* **2009**, *101*, 569-577, doi:10.1016/s0027-9684(15)30942-1.

57. Vesco, K.K.; Karanja, N.; King, J.C.; Gillman, M.W.; Leo, M.C.; Perrin, N.; McEvoy, C.T.; Eckhardt, C.L.; Smith, K.S.; Stevens, V.J. Efficacy of a group-based dietary intervention for limiting gestational weight gain among obese women: a randomized trial. *Obesity (Silver Spring)* **2014**, *22*, 1989-1996, doi:10.1002/oby.20831.

58. Vinter, C.A.; Jensen, D.M.; Ovesen, P.; Beck-Nielsen, H.; Jørgensen, J.S. The LiP (Lifestyle in Pregnancy) study: a randomized controlled trial of lifestyle intervention in 360 obese pregnant women. *Diabetes Care* **2011**, *34*, 2502-2507, doi:10.2337/dc11-1150.

59. Beauchesne, A.R.; Cara, K.C.; Chen, J.; Yao, Q.; Penkert, L.P.; Yang, W.; Chung, M. Effectiveness of multimodal nutrition interventions during pregnancy to achieve 2009 Institute of Medicine gestational weight gain guidelines: a systematic review and meta-analysis. *Ann Med* **2021**, *53*, 1179-1197, doi:10.1080/07853890.2021.1947521.

60. Wolff, S.; Legarth, J.; Vangsgaard, K.; Toubro, S.; Astrup, A. A randomized trial of the effects of dietary counseling on gestational weight gain and glucose metabolism in obese pregnant women. *Int J Obes (Lond)* **2008**, *32*, 495-501, doi:10.1038/sj.ijo.0803710.

**Supplementary Table 6** Relative effect sizes of treatments efficacy at post-treatment.

| 93.8% |  | High probability of being the best intervention (with high SUCRA values) | |  |  |  |  |  |
| --- | --- | --- | --- | --- | --- | --- | --- | --- |
| I-D | 74.7% |  |  |  |  |  | **Active Interventions** | |
| -0.68  (-1.80,0.45) | I-PADB | 72.3% |  |  |  |  | **Significant pairwise comparisons** | |
| -0.68  (-1.90,0.55) | 0.00  (0.96,0.96) | I-B+R-B(m) | 67.5% |  |  |  | **SUCRA value** | |
| -0.77  (-1.98,0.43) | -0.10  (-1.04,0.85) | -0.10  (-1.16,0.96) | R-PADB(e) | 63.9% |  |  |  |  |
| -0.79  (-2.20,0.63) | -0.11  (-1.27,1.06) | -0.11  (-1.40,1.18) | -0.01  (-1.29,1.27) | I-E+R-PA(m) | 63.2% |  | High probability of being the worst intervention (with low SUCRA values) | |
| -0.87  (-1.92,0.18) | -0.19  (-0.92,0.53) | -0.20  (-1.07,0.68) | -0.10  (-0.95,0.76) | -0.09  (-1.20,1.02) | I-PAB | 62.4% |  |  |
| -0.88  (-2.01,0.26) | -0.20  (-1.03,0.63) | -0.20  (-1.18,0.78) | -0.10  (-1.06,0.86) | -0.09  (-1.25,1.07) | -0.00  (-0.74,0.74) | I-PAD | 62.1% |  |
| -0.75  (-2.58,1.09) | -0.07  (-1.75,1.61) | -0.07  (-1.82,1.68) | 0.03  (-1.66,1.71) | 0.04  (-1.85,1.93) | 0.13  (-1.51,1.76) | 0.13  (-1.56,1.82) | R-PADE(e) | 61.3% |
| -0.86  (-2.10,0.37) | -0.19  (-1.15,0.78) | -0.19  (-1.27,0.90) | -0.09  (-1.16,0.99) | -0.08  (-1.38,1.23) | 0.01  (-0.88,0.90) | 0.01 (-0.99,1.01) | -0.12  (-1.87,1.64) | R-PADB(m) |
| -0.93  (-1.92,0.05) | -0.26  (-0.86,0.34) | -0.26  (-1.03,0.52) | -0.16  (-0.93,0.61) | -0.15  (-1.21,0.92) | -0.06  (-0.54,0.42) | -0.06  (-0.71,0.60) | -0.19  (-1.78,1.40) | -0.07  (-0.84,0.70) |
| -1.05  (-2.11,0.02) | -0.37  (-1.13,0.38) | -0.37  (-1.27,0.52) | -0.27  (-1.15,0.60) | -0.26  (-1.41,0.89) | -0.18  (-0.82,0.47) | -0.17 (-0.95,0.61) | -0.30  (-1.94,1.34) | -0.18  (-1.10,0.73) |
| -1.06  (-2.46,0.34) | -0.38  (-1.56,0.80) | -0.38  (-1.66,0.89) | -0.29 (-1.54,0.97) | -0.27  (-1.73,1.19) | -0.19  (-1.30,0.92) | -0.18  (-1.38,1.01) | -0.31  (-2.19,1.56) | -0.20  (-1.48,1.09) |
| -1.10  (-2.51,0.30) | -0.43  (-1.62,0.77) | -0.43  (-1.71,0.86) | -0.33  (-1.60,0.94) | -0.32  (-1.79,1.15) | -0.23  (-1.35,0.89) | -0.23 (-1.43,0.98) | -0.36  (-2.24,1.52) | -0.24  (-1.54,1.06) |
| -1.13  (-2.65,0.39) | -0.45  (-1.74,0.84) | -0.45  (-1.85,0.95) | -0.35 (-1.74,1.04) | -0.34  (-1.84,1.16) | -0.25  (-1.49,0.99) | -0.25  (-1.53,1.03) | -0.38  (-2.34,1.59) | -0.26  (-1.68,1.15) |
| -1.13  (-2.29,0.03) | -0.45  (-1.34,0.43) | -0.46  (-1.47,0.55) | -0.36  (-1.35,0.63) | -0.34  (-1.58,0.89) | -0.26  (-1.05,0.53) | -0.26  (-1.16,0.65) | -0.38  (-2.09,1.32) | -0.27  (-1.29,0.76) |
| -1.15  (-2.55,0.25) | -0.47  (-1.61,0.67) | -0.47  (-1.75,0.80) | -0.38  (-1.63,0.88) | -0.36  (-1.74,1.01) | -0.28  (-1.36,0.81) | -0.27  (-1.41,0.86) | -0.40  (-2.28,1.47) | -0.29  (-1.57,1.00) |
| -1.16  (-2.63,0.31) | -0.48  (-1.75,0.78) | -0.49  (-1.84,0.87) | -0.39  (-1.72,0.95) | -0.37  (-1.90,1.16) | -0.29  (-1.49,0.91) | -0.29 (-1.56,0.99) | -0.41  (-2.34,1.51) | -0.30  (-1.66,1.07) |
| -1.13  (-2.15, -0.10) | -0.45  (-1.14,0.24) | -0.45  (-1.30,0.40) | -0.35  (-1.17,0.47) | -0.34  (-1.43,0.75) | -0.25  (-0.82,0.31) | -0.25  (-0.90,0.40) | -0.38  (-1.99,1.24) | -0.26  (-1.13,0.60) |
| -1.14 (-2.17, -0.12) | -0.47  (-1.17,0.24) | -0.47  (-1.32,0.38) | -0.37  (-1.09,0.35) | -0.36  (-1.47,0.75) | -0.27  (-0.85,0.31) | -0.27  (-0.99,0.46) | -0.40  (-1.92,1.13) | -0.28  (-1.15,0.59) |
| -1.18  (-2.72,0.36) | -0.51  (-1.85,0.84) | -0.51  (-1.94,0.92) | -0.41  (-1.77,0.95) | -0.40  (-1.99,1.20) | -0.31  (-1.60,0.97) | -0.31  (-1.67,1.05) | -0.44  (-1.44,0.57) | -0.32  (-1.76,1.12) |
| -1.21  (-2.22, -0.19) | -0.53  (-1.15,0.09) | -0.53  (-1.36,0.31) | -0.43  (-1.24,0.38) | -0.42  (-1.40,0.57) | -0.33  (-0.84,0.18) | -0.33  (-0.94,0.28) | -0.46  (-2.07,1.15) | -0.34  (-1.19,0.51) |
| -1.32  (-2.80,0.17) | -0.64  (-1.89,0.61) | -0.64  (-2.01,0.73) | -0.54  (-1.90,0.81) | -0.53  (-1.99,0.94) | -0.44  (-1.64,0.76) | -0.44  (-1.68,0.80) | -0.57  (-2.51,1.37) | -0.45  (-1.83,0.93) |
| -1.29 (-2.52, -0.07) | -0.62  (-1.58,0.34) | -0.62  (-1.69,0.46) | -0.52  (-1.58,0.54) | -0.51  (-1.80,0.79) | -0.42  (-1.30,0.46) | -0.42 (-1.40,0.56) | -0.55  (-2.29,1.20) | -0.43  (-1.20,0.34) |
| -1.42 (-2.83, -0.02) | -0.75  (-1.93,0.44) | -0.75  (-2.03,0.54) | -0.65  (-1.91,0.62) | -0.64  (-2.10,0.83) | -0.55  (-1.67,0.57) | -0.55  (-1.75,0.65) | -0.68  (-2.55,1.20) | -0.56  (-1.85,0.73) |
| -1.27  (-2.23, -0.32) | -0.60  (-1.19, -0.00) | -0.60  (-1.36,0.17) | -0.50 (-1.23,0.24) | -0.49  (-1.53,0.56) | -0.40  (-0.84,0.04) | -0.40  (-1.02,0.22) | -0.53  (-2.10,1.04) | -0.41  (-1.19,0.38) |
| -1.64 (-3.14, -0.15) | -0.97  (-2.23,0.29) | -0.97  (-2.35,0.41) | -0.87  (-2.23,0.49) | -0.86  (-2.33,0.62) | -0.77  (-1.98,0.44) | -0.77  (-2.02,0.48) | -0.90  (-2.84,1.05) | -0.78  (-2.17,0.61) |

*Continued...*

| 59.1% |  | High probability of being the best intervention (with high SUCRA values) | |  |  |  |  |
| --- | --- | --- | --- | --- | --- | --- | --- |
| I-B | 49.2% |  |  |  |  |  |  |
| -0.11  (-0.63,0.40) | I-DB | 48.2% |  |  |  | High probability of being the worst intervention (with low SUCRA values) | |
| -0.12  (-1.17,0.92) | -0.01  (-1.14,1.11) | R-PA(m+e) | 45.3% |  |  |  |  |
| -0.17  (-1.23,0.89) | -0.06  (-1.19,1.08) | -0.04  (-1.50,1.41) | I-PADB+RB(e) | 44.7% |  |  |  |
| -0.19  (-1.39,1.01) | -0.08  (-1.35,1.19) | -0.07  (-1.63,1.49) | -0.02  (-1.59,1.55) | I-PAE | 43.2% |  |  |
| -0.20  (-0.90,0.50) | -0.08  (-0.90,0.73) | -0.07  (-1.29,1.14) | -0.03  (-1.25,1.20) | -0.01  (-1.36,1.35) | I-B+RB(e) | 42.9% |  |
| -0.21  (-1.25,0.82) | -0.10  (-1.23,1.02) | -0.09  (-1.53,1.35) | -0.05  (-1.50,1.41) | -0.02  (-1.51,1.46) | -0.02  (-1.23,1.20) | I-B+R-DB(e) | 42.9% |
| -0.23  (-1.37,0.91) | -0.11  (-1.33,1.10) | -0.10  (-1.61,1.41) | -0.06  (-1.58,1.46) | -0.04  (-1.66,1.59) | -0.03  (-1.33,1.27) | -0.01  (-1.52,1.50) | I-DB+R-B(e) |
| -0.19  (-0.63,0.24) | -0.08  (-0.68,0.52) | -0.07  (-1.15,1.02) | -0.02  (-1.12,1.08) | 0.00  (-1.22,1.22) | 0.01  (-0.75,0.76) | 0.02  (-1.04,1.09) | 0.04  (-1.14,1.21) |
| -0.21  (-0.65,0.23) | -0.10  (-0.70,0.51) | -0.08  (-1.17,1.00) | -0.04  (-1.14,1.06) | -0.02  (-1.26,1.22) | -0.01  (-0.77,0.75) | 0.01  (-1.08,1.09) | 0.02  (-1.16,1.20) |
| -0.25  (-1.48,0.98) | -0.14  (-1.43,1.16) | -0.12  (-1.71,1.46) | -0.08  (-1.67,1.51) | -0.06  (-1.75,1.63) | -0.05  (-1.43,1.33) | -0.03  (-1.62,1.55) | -0.02  (-1.67,1.62) |
| -0.27  (-0.67,0.13) | -0.16  (-0.74,0.43) | -0.15  (-1.22,0.93) | -0.10  (-1.19,0.99) | -0.08  (-1.21,1.05) | -0.07  (-0.82,0.67) | -0.06  (-1.01,0.90) | -0.04  (-1.21,1.13) |
| -0.38  (-1.54,0.78) | -0.27  (-1.50,0.97) | -0.26  (-1.79,1.27) | -0.21  (-1.75,1.33) | -0.19  (-1.75,1.38) | -0.18  (-1.50,1.13) | -0.17  (-1.61,1.28) | -0.15  (-1.75,1.44) |
| -0.36  (-1.14,0.42) | -0.25  (-1.14,0.65) | -0.23  (-1.51,1.04) | -0.19  (-1.48,1.10) | -0.17  (-1.57,1.24) | -0.16  (-1.17,0.85) | -0.14  (-1.42,1.13) | -0.13  (-1.49,1.22) |
| -0.49  (-1.54,0.57) | -0.37  (-1.51,0.76) | -0.36  (-1.81,1.09) | -0.32  (-1.78,1.14) | -0.30  (-1.86,1.27) | -0.29  (-1.51,0.93) | -0.27  (-1.72,1.18) | -0.26  (-1.78,1.26) |
| -0.34  (-0.57, -0.10) | -0.23  (-0.70,0.25) | -0.21  (-1.23,0.81) | -0.17  (-1.20,0.86) | -0.15  (-1.33,1.03) | -0.14  (-0.80,0.52) | -0.12  (-1.14,0.90) | -0.11  (-1.23,1.01) |
| -0.71  (-1.88,0.46) | -0.60  (-1.84,0.65) | -0.58  (-2.12,0.95) | -0.54  (-2.09,1.00) | -0.52  (-2.09,1.05) | -0.51  (-1.84,0.81) | -0.49  (-1.95,0.96) | -0.48  (-2.09,1.12) |

| 42.5% |  | High probability of being the best intervention (with high SUCRA values) | |  |  |  |  |  |
| --- | --- | --- | --- | --- | --- | --- | --- | --- |
| I-PA | 40.7% |  |  |  |  |  |  |  |
| -0.02  (-0.55,0.51) | R-B(e) | 40.7% |  |  |  |  |  |  |
| -0.06  (-1.32,1.21) | -0.04  (-1.19,1.11) | R-E(e) | 35.4% |  |  |  |  |  |
| -0.08  (-0.54,0.38) | -0.06  (-0.57,0.45) | -0.02  (-1.28,1.24) | I-E | 34.4% |  |  |  |  |
| -0.19  (-1.37,0.99) | -0.17 (-1.37,1.03) | -0.13  (-1.79,1.53) | -0.11  (-1.9,0.97) | I-PADB+R-PAB(e) | 32.5% |  | High probability of being the best intervention (with high SUCRA values) | |
| -0.17  (-1.02,0.68) | -0.15  (-1.00,0.70) | -0.11  (-1.54,1.32) | -0.09 (-0.93,0.75) | 0.02  (-1.35,1.39) | R-B(m) | 28.7% |  |  |
| -0.30  (-1.39,0.80) | -0.28  (-1.37,0.82) | -0.24  (-1.83,1.35) | -0.22  (-1.30,0.87) | -0.11  (-1.64,1.43) | -0.13 (-1.41,1.15) | R-B(m+e) | 28.2% |  |
| -0.15  (-0.52,0.22) | -0.13  (-0.51,0.25) | -0.09 (-1.30,1.12) | -0.07  (-0.42,0.28) | 0.04  (-1.10,1.18) | 0.02  (-0.75,0.79) | 0.15  (-0.88,1.18) | Placebo | 19.9% |
| -0.52  (-1.71,0.67) | -0.50  (-1.71,0.71) | -0.46  (-2.13,1.21) | -0.44  (-1.53,0.66) | -0.33  (-1.87,1.21) | -0.35  (-1.73,1.03) | -0.22  (-1.76,1.32) | -0.37  (-1.52,0.78) | R-PAB(e) |

*Continued…*
